# Effects of High-Intensity Interval Training on Body Composition, Metabolic Health, and Cardiorespiratory Fitness in Overweight or Obese Children and Adolescents: A Systematic Review and Meta-Analysis

**DOI:** 10.3390/metabo16040232

**Published:** 2026-03-31

**Authors:** Yao Yan, Cheng Peng, Hongjun Zhang, Biaoxu Tao, Shuning Liu, Shuairan Li, Jing Mi, Chang Liu

**Affiliations:** 1 China Basketball College, Beijing Sport University, Beijing 100084, China; 2024210467@bsu.edu.cn; 2 Sports Coaching College, Beijing Sport University, Beijing 100084, China; pengchengbsu@163.com (C.P.); 2025110035@bsu.edu.cn (H.Z.); 2022110017@bsu.edu.cn (S.L.); 3 School of Sports Training, Wuhan Sports University, Wuhan 430079, China; 2024410681@whsu.edu.cn; 4 School of Sport Science, Beijing Sport University, Beijing 100084, China; 2023013553@bsu.edu.cn

**Keywords:** high-intensity interval training, overweight, obesity, children, adolescents, body composition, metabolic health, cardiorespiratory fitness, systematic review, meta-analysis

## Abstract

**Background**: Childhood and adolescent overweight and obesity are major global public health concerns. High-intensity interval training (HIIT) has been increasingly investigated as a time-efficient intervention; however, evidence regarding its effects on multiple health-related outcomes and the influence of intervention characteristics remains inconsistent. **Objective**: The objective of this study was to evaluate the effects of HIIT on body composition, metabolic health, and cardiorespiratory fitness in children and adolescents with overweight or obesity. **Methods**: Systematic literature searches were conducted in PubMed, Web of Science, EBSCO, CNKI, Wanfang Data, and VIP databases. Randomized controlled trials were included. Risk of bias was assessed using the Cochrane Risk of Bias tool. Meta-analyses were conducted using random-effects models, and standardized mean differences (SMDs) with 95% confidence intervals (CIs) were calculated. Heterogeneity, sensitivity analyses, publication bias, and certainty of evidence (GRADE) were also evaluated. **Results**: A total of 21 randomized controlled trials involving 652 participants (325 in the intervention groups and 327 in the control groups) were included. Compared with control conditions, HIIT significantly improved multiple outcomes related to body composition, metabolic health, and cardiorespiratory fitness, including BMI (SMD = −1.05), body fat percentage (SMD = −0.69), total cholesterol (SMD = −0.42), HOMA-IR (SMD = −1.00), and VO_2_peak (SMD = 0.91), while no significant effect was observed on lean body mass. Subgroup analyses suggested that HIIT protocols with a load duration of less than 1 min were associated with greater improvements in several outcomes, particularly body fat percentage, total cholesterol, HOMA-IR, and VO_2_peak. **Conclusions**: HIIT may improve body composition, metabolic health, and cardiorespiratory fitness in children and adolescents with overweight or obesity. However, the certainty of evidence varied across outcomes and was limited for some findings by heterogeneity, small sample sizes, and potential risk of bias. Further high-quality, large-scale randomized controlled trials with standardized HIIT protocols are needed to confirm these findings and clarify the influence of different training characteristics (e.g., exercise mode and interval structure).

## 1. Introduction

Overweight and obesity in childhood and adolescence have become major public health challenges worldwide. According to the World Health Organization, more than 390 million children and adolescents aged 5–19 years were overweight or obese in 2022, and the global prevalence in this age group increased from 8% in 1990 to 20% in 2022 [1]. A recent global analysis further showed that, in many countries, obesity in school-aged children and adolescents has now surpassed thinness, underscoring the worldwide shift in malnutrition patterns [2]. For example, obesity affects 19.7% of children and adolescents aged 2–19 years in the United States [3], while data from the WHO European Childhood Obesity Surveillance Initiative (COSI) show that 28.7% of boys and 26.5% of girls aged 6–9 years across 36 countries were overweight or obese [4]. Importantly, this burden is not confined to a single country or to high-income settings. Consistent with these global trends, China has also experienced a marked increase in overweight and obesity among children and adolescents over recent decades [5]. The *Report on the Status of Nutrition and Chronic Diseases Among Chinese Residents (2020)* issued by the National Health Commission indicates that the prevalence of overweight and obesity among children and adolescents aged 6–17 years has reached 19% and continues to show an upward trend [6]. Childhood and adolescent obesity not only impairs physical development and mental well-being but also markedly increases the risk of insulin resistance, dyslipidemia, hypertension, and early cardiovascular and metabolic complications, posing a serious threat to long-term health [7].

Exercise-based interventions are among the most effective and safest non-pharmacological strategies for managing pediatric overweight and obesity. In recent years, high-intensity interval training (HIIT), which alternates brief bouts of vigorous exercise with low-intensity recovery, has attracted increasing attention as a time-efficient modality that may improve body composition and cardiometabolic health while also promoting adherence and enjoyment [8,9,10].

Previous systematic reviews and meta-analyses have suggested that HIIT may improve selected health-related outcomes in children and adolescents with overweight or obesity, particularly cardiorespiratory fitness and some cardiometabolic risk markers [11,12,13]. However, the current evidence base remains incomplete. Some earlier meta-analyses primarily focused on comparisons between HIIT and moderate-intensity continuous training and emphasized cardiometabolic risk factors rather than the overall effects of HIIT across multiple outcome domains [11,12]. In contrast, other more recent reviews have focused on a single outcome, such as cardiorespiratory fitness, rather than simultaneously evaluating body composition, metabolic health, and cardiorespiratory fitness within one integrated synthesis [13]. In addition, findings from individual randomized controlled trials have not been fully consistent, with the magnitude of improvement varying across studies and outcomes [11,12]. These discrepancies may be related to heterogeneity in participant characteristics, intervention duration, exercise modality, work-to-rest structure, and outcome selection [11,12,13]. Therefore, an updated and more comprehensive synthesis is warranted to clarify the overall effects of HIIT and to explore whether key intervention characteristics may influence its effectiveness.

Accordingly, we conducted a systematic review and meta-analysis to synthesize the current evidence on the effects of HIIT on body composition, metabolic health, and cardiorespiratory fitness in children and adolescents with overweight or obesity. We further explored whether selected intervention characteristics, such as load duration and training modality, might be associated with differences in intervention effects, thereby informing exercise-based interventions and public health strategies for pediatric overweight and obesity.

## 2. Methods

This study followed the Preferred Reporting Items for Systematic Reviews and Meta-Analyses (PRISMA) guidelines [14] and was registered on the PROSPERO database, the registration No. is CRD420251155266.

### 2.1. PICOS and Literature Search Strategy

The search strategy was developed based on the Population, Intervention, Comparison, Outcome, and Study design (PICOS) framework (Table 1). Eligible participants were children and adolescents with overweight or obesity, without restrictions on sex, nationality, or region.

A comprehensive literature search was conducted across several databases, including Web of Science, PubMed, EBSCO, CNKI, Wanfang Data, and VIP from their inception to 9 September 2025. The search strategy was developed using a combination of Medical Subject Headings (MeSH) and free-text terms to ensure comprehensive retrieval of relevant studies. The English search terms included but were not limited to “high-intensity interval training”, “high-intensity interval exercise”, “high-intensity intermittent exercise”, “adolescent”, “children”, “childhood”, “overweight”, and “obese”. Boolean operators (“AND” and “OR”) were used to combine related terms. The complete search strategies, including the Boolean search strings used for each database, are provided in Supplementary Material S1.

### 2.2. Inclusion and Exclusion Criteria

#### Inclusion Criteria

The inclusion criteria determined whether the included studies were relevant to the scope of this study and ultimately affected the validity of the findings. Therefore, we predefined the inclusion criteria based on the PICOS framework. Moreover, to include as many potentially eligible studies as possible, we imposed no time limitations on the literature search. Specific inclusion criteria were as follows: (1) Participants were children and adolescents aged between 5 and 19 years, according to the WHO Growth Reference [15], and were classified as overweight or obese based on the criteria established by the World Health Organization or relevant national standards, without other contraindications to exercise or health problems. (2) The intervention group received HIIT, while the control group maintained usual activities without additional structured exercise intervention. (3) Outcomes included at least one measure related to body composition, metabolic health, or cardiorespiratory fitness. (4) The study design was a randomized controlled trial (RCT). (5) The study was published in Chinese or English.

### 2.3. Exclusion Criteria

Conversely, the exclusion criteria were as follows:

(1) Studies enrolling normal-weight participants in control groups;
(2) Ineligible study types (e.g., literature reviews, conference abstracts, animal studies, acute trials, or single-arm studies);
(3) Studies without accessible full texts or extractable data;
(4) Non-peer-reviewed publications (e.g., dissertations, theses, preprints).

### 2.4. Study Selection

The literature search records were managed and screened using Zotero software (Version 7.0.17; Corporation for Digital Scholarship, Falls Church, VA, USA, 2025). Two independent reviewers (Y.Y. and C.P.) first removed duplicate records. Titles and abstracts were then screened according to the predefined inclusion and exclusion criteria to identify potentially eligible studies. The full texts of the remaining articles were subsequently reviewed to determine final eligibility. Any disagreements arising during this process were resolved by discussion, and if consensus could not be achieved, a third reviewer (H.J.Z.) was consulted for adjudication.

### 2.5. Data Extraction

Data were independently extracted by the same two reviewers. The extracted information included: (1) the first author and year of publication; (2) participant characteristics; (3) sample size; (4) intervention details including intervention duration, frequency, session length, exercise modality, and intervention protocol; and (5) outcome measures.

For continuous outcomes, effect sizes were calculated using post-intervention values rather than change scores. Because baseline values were generally comparable between the intervention and control groups in the randomized trials, post-intervention data were considered appropriate for estimating between-group effects.

For the calculation of effect sizes, the mean values and standard deviations (SDs) were extracted for both intervention and control groups. When these data were not directly reported, other statistics (e.g., medians with ranges or standard errors) were used to calculate the corresponding values using appropriate formulas.

For studies with multiple intervention arms, only the HIIT arms were included in the quantitative synthesis. When multiple time points were reported, only the assessment conducted immediately after completion of the intervention was extracted for the primary meta-analysis; follow-up assessments were not included.

### 2.6. Quality Assessment

To assess the methodological quality of the RCTs included in this study, the Cochrane Risk of Bias (RoB) tool, implemented in Review Manager (Version 5.4; The Cochrane Collaboration, 2020), was used by two of our researchers (Y.Y. and H.J.Z.) independently. This tool evaluates potential risk of bias in these studies across six domains: selection, performance, detection, attrition, reporting, and other sources of bias. Specifically, seven items were assessed: random sequence generation, allocation concealment, blinding of participants and personnel, blinding of outcome assessment, incomplete outcome data, selective reporting, and other bias. Each item was rated as having a “low risk,” “high risk,” or “unclear risk” of bias.

### 2.7. Statistical Analysis

Statistical analyses were performed using Stata/MP version 18.0 (StataCorp LLC, College Station, TX, USA). For continuous outcomes, pooled effect sizes were calculated as standardized mean differences (SMDs) with 95% confidence intervals (CIs), because the included studies used different measurement scales. Given the relatively small sample sizes of the included studies, Hedges’ g was applied to correct for small-sample bias; for interpretability, effect sizes were classified as small (0.20–0.49), medium (0.50–0.79), or large (≥0.80) [16].

Statistical heterogeneity was assessed using Cochran’s Q test and quantified using the I^2^ statistic. I^2^ values of 25%, 50%, and 75% were interpreted as indicating low, moderate, and high heterogeneity, respectively [17].

Regarding model choice, a fixed-effect model assumes a single common true effect across studies. When heterogeneity is present, this assumption may be violated because the model does not account for between-study variance (τ^2^), which can lead to underestimated uncertainty and overly narrow confidence intervals; moreover, inference pertains only to the specific set of included studies [18,19]. Given the anticipated clinical and methodological heterogeneity in participant characteristics and HIIT prescriptions across trials, pooled effect sizes were therefore synthesized using a random-effects model. This model incorporates between-study variance (τ^2^) and estimates the mean of a distribution of true effects, thereby providing a more appropriate summary of the available evidence under heterogeneous conditions [18,19].

Sensitivity analyses were conducted using a leave-one-out approach, in which each study was sequentially omitted and the pooled estimate was recalculated to assess the robustness of the results. For outcomes included in publication bias assessment, potential small-study effects were examined using visual inspection of funnel plots and Egger’s regression test [20,21]. If Egger’s test indicated possible bias (*p* < 0.05), the trim-and-fill method was applied to adjust for missing studies, and the corrected pooled estimates were recalculated to verify the robustness of these outcomes. Given the limited number of studies for some outcomes and the incomplete reporting of potential effect modifiers, only prespecified subgroup analyses were conducted, whereas more extensive subgroup analyses or meta-regression were not performed to avoid underpowered and unstable estimates.

### 2.8. Certainty Assessment

To assess how much confidence can be placed in the pooled effects for each outcome, we used the GRADE approach to rate the certainty of evidence. This approach evaluates five domains for each outcome: risk of bias, inconsistency, indirectness, imprecision, and publication bias. As all included studies were randomized controlled trials, the starting level was “high”, and if any important concerns were identified in these domains, the rating was downgraded to moderate, low, or very low. The GRADE assessment was performed by one reviewer (Y.Y.), with independent verification by a second reviewer (C.P.).

## 3. Results

### 3.1. Literature Search Results

**Figure 1:**
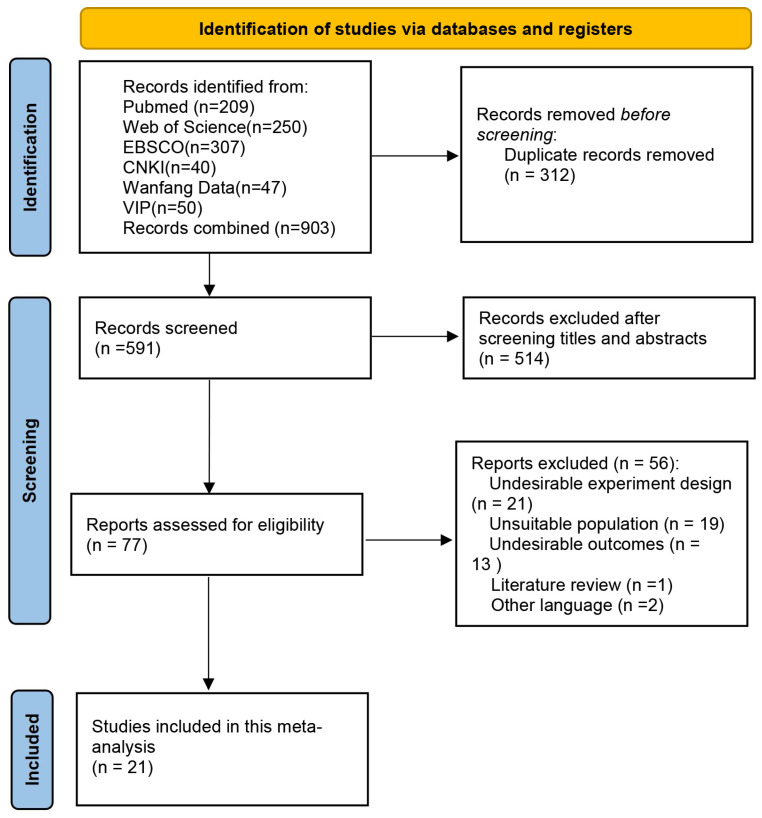
Flow diagram of included and excluded studies.

Following the search strategy described above, a total of 903 records were retrieved from the selected databases. After removing duplicates using Zotero, 591 unique records remained. Subsequently, titles and abstracts were screened according to the predefined inclusion and exclusion criteria, and clearly irrelevant studies were removed, resulting in 77 articles for full-text review. After full-text assessment, 21 studies met the eligibility criteria and were included in the final analysis [22,23,24,25,26,27,28,29,30,31,32,33,34,35,36,37,38,39,40,41,42]. The study selection process is summarized in Figure 1.

### 3.2. Study Characteristics

A total of 903 publications were initially identified through the database search. After removing 312 duplicate records, 591 articles remained for title and abstract screening, of which 514 were excluded. Subsequently, the full texts of 77 articles were assessed for eligibility. Ultimately, 21 studies were included in this meta-analysis, with a total of 652 participants (325 in the intervention groups and 327 in the control groups). Table 2 presents the main characteristics of the included studies.

### 3.3. Study Quality Assessment

**Figure 2:**
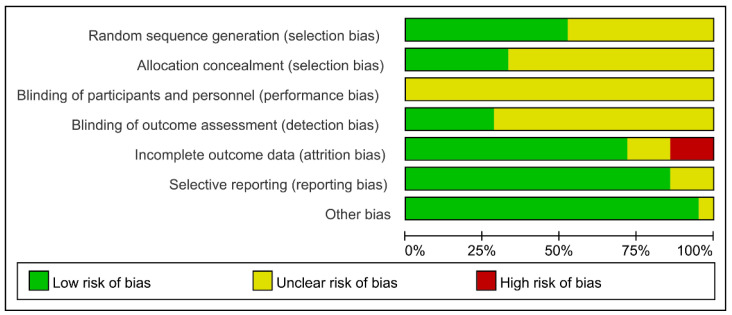
Risk of bias graph.

**Figure 3:**
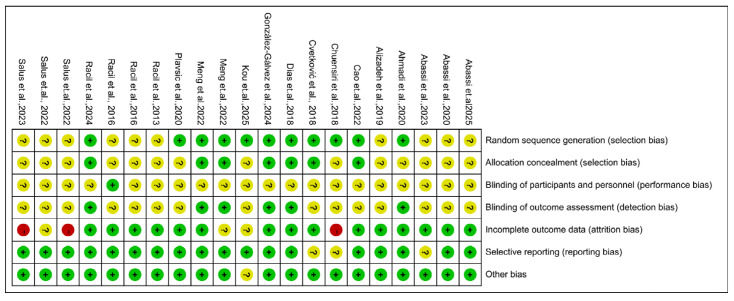
Risk of bias summary of the included studies [22,23,24,25,26,27,28,29,30,31,32,33,34,35,36,37,38,39,40,41,42].

We utilized the RoB tool implemented in Review Manager (Version 5.4; The Cochrane Collaboration, 2020) to evaluate the methodological quality of the 21 included studies. Although all included studies were randomized controlled trials, 10 studies did not explicitly describe how the randomization sequence was generated; therefore, the risk of bias for random sequence generation in these studies was rated as “unclear”. Six studies explicitly described their allocation concealment procedures; for the remaining trials, concealment methods were not reported, and this domain was rated as “unclear” risk of bias. None of the included studies reported a specific method for blinding participants and study personnel, and given the nature of exercise interventions, performance bias in this domain could not be excluded. The risk of bias related to blinding of outcome assessment also varied across studies. In addition, the risk of bias arising from incomplete outcome data and selective reporting was not consistent among the included trials; depending on attrition, exclusions after randomization, and the availability of trial registration or protocol information, some studies were judged as having low risk, whereas others were rated as having high or unclear risk. The overall results of the quality assessment are presented in Figure 2 and Figure 3.

### 3.4. Results of Meta-Analysis

#### 3.4.1. Outcomes of Body Composition

A meta-analysis of 15 studies indicated that, compared with the control conditions, HIIT had a significant overall effect on reducing BMI in children and adolescents with overweight or obesity (SMD = −1.05, 95% CI [−1.56, −0.55], *p* < 0.001). According to our pre-specified benchmarks for Hedges’ g, the magnitude of this pooled effect qualifies as large. Although between-study heterogeneity was substantial (I^2^ = 84%), sensitivity analysis showed that exclusion of any single study did not materially change the pooled estimate, and all leave-one-out re-estimates remained negative with 95% CIs below zero, indicating a robust overall effect despite between-study variability.

A meta-analysis of 13 studies revealed that HIIT had a significant overall effect on reducing waist circumference (WC) in children and adolescents with overweight or obesity (SMD = −0.38, 95% CI [−0.61, −0.16], *p* = 0.01), and the magnitude of this pooled effect was small. Heterogeneity across studies was low (I^2^ = 20%), suggesting high consistency of outcomes among individual studies. Sensitivity analysis also indicated that the pooled effect was robust.

A meta-analysis of 18 studies showed that, compared with the control conditions, HIIT significantly reduced body fat percentage (BF%) in children and adolescents with overweight or obesity, and the effect size was medium (SMD = −0.69, 95% CI [−1.01, −0.37], *p* < 0.001). Heterogeneity across studies was moderate (I^2^ = 67%), suggesting a moderate level of variability between studies. The robustness of the overall effect was confirmed by a leave-one-out sensitivity analysis.

A meta-analysis of seven studies found that, compared with the control conditions, HIIT was associated with a reduction in fat mass (FM) (SMD = −0.89, 95% CI [−1.59, −0.19], *p* = 0.012), which represents a large effect. Substantial heterogeneity was observed among studies (I^2^ = 83%). The leave-one-out sensitivity analysis supported the robustness of the pooled effect, as removing any single study did not materially alter its magnitude or direction.

**Figure 4:**
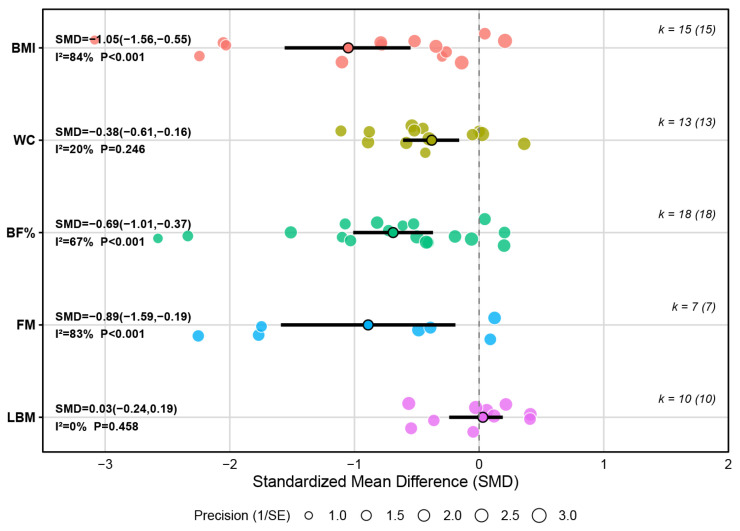
Summary of the impact of HIIT on body composition.

However, a meta-analysis of 10 studies found that, compared with the control conditions, HIIT had no statistically significant effect on lean body mass (LBM) in children and adolescents with overweight or obesity (SMD = −0.03, 95% CI [−0.24, 0.19], *p* = 0.813). More details can be found in Figure 4 and Figure S1 in Supplementary Material S2.

#### 3.4.2. Outcomes of Metabolic Health

A meta-analysis of 9 studies showed that, compared with the control conditions, HIIT had a significant overall effect on reducing total cholesterol (TC) in children and adolescents with overweight or obesity (SMD = −0.42, 95% CI [−0.66, −0.17], *p* = 0.001), representing a small effect size. Heterogeneity was low (I^2^ = 10%), suggesting a high consistency of outcomes among included studies. Sensitivity analysis indicated that excluding any single study did not materially change the pooled estimate, indicating a robust overall effect.

A meta-analysis of nine studies showed that, compared with control conditions, HIIT had a significant overall effect on increasing high-density lipoprotein cholesterol (HDL-C) in children and adolescents with overweight or obesity (SMD = 0.45, 95% CI [0.08, 0.81], *p* = 0.016), representing a small-to-medium effect size. The between-study heterogeneity was moderate (I^2^ = 57%), and sensitivity analysis showed that exclusion of any single study did not materially change the pooled estimate, indicating a robust overall effect despite potential between-study variability.

A meta-analysis of nine studies showed that, compared with control conditions, HIIT had a significant overall effect on decreasing low-density lipoprotein cholesterol (LDL-C) in children and adolescents with overweight or obesity (SMD = −0.58, 95% CI [−0.94, −0.21], *p* = 0.002), representing a medium effect size. The heterogeneity between-study was moderate (I^2^ = 58%), and sensitivity analysis revealed the robustness of the overall effect.

A meta-analysis of nine studies indicated that, compared with control conditions, HIIT had a significant overall effect on decreasing triglyceride (TG) levels in children and adolescents with overweight or obesity (SMD = −0.32, 95% CI [−0.59, −0.05], *p* = 0.02), representing a small effect size. Outcomes between studies were relatively consistent as heterogeneity was low (I^2^ = 26%). Sensitivity analysis found that excluding any single study did not materially alter the pooled estimate, indicating a robust overall effect despite between-study variability.

A meta-analysis of eight studies suggested that, compared with control conditions, HIIT had a significant overall effect on reducing insulin levels in children and adolescents with overweight or obesity (SMD = −1.58, 95% CI [−2.09, −1.08], *p* < 0.001), representing a large effect size. The heterogeneity between-study was moderate (I^2^ = 62%), and sensitivity analysis showed that exclusion of any single study did not materially change the pooled estimate, indicating a robust overall effect.

A meta-analysis of 10 studies suggested that, compared with control conditions, HIIT had a significant overall effect on decreasing glucose levels in children and adolescents with overweight or obesity (SMD = −0.53, 95% CI [−0.96, −0.09], *p* = 0.017), representing a medium effect size. Although between-study heterogeneity indicated variability across studies (I^2^ = 68%), sensitivity analysis indicated a robust pooled effect, with exclusion of any single study causing no material change in the estimate.

**Figure 5:**
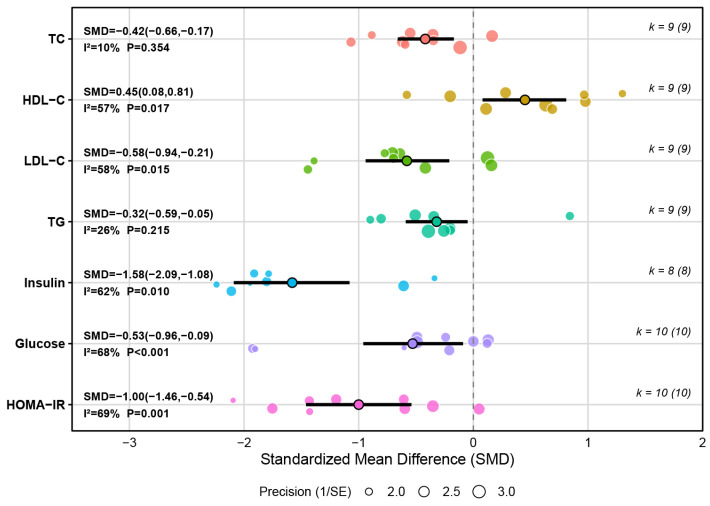
Summary of the impact of HIIT on metabolic health.

A meta-analysis of 10 studies suggested that, compared with control conditions, HIIT had a significant overall effect on reducing HOMA-IR in children and adolescents with overweight or obesity (SMD = −1.00, 95% CI [−1.46, −0.54], *p* < 0.001), indicating a large effect size. While the heterogeneity between-studies was moderate (I^2^ = 69%), sensitivity analysis revealed that exclusion of any single study did not materially change the pooled estimate, indicating a robust overall effect despite between-study variability. More details could be found in Figure 5 and Figure S2 in Supplementary Material S2.

#### 3.4.3. Outcomes of Cardiorespiratory Fitness

A total of 10 studies examined the effects of HIIT on systolic blood pressure (SBP), including 284 participants. The results showed that HIIT had a significant effect on reducing SBP in children and adolescents with overweight and obesity compared with the control conditions (SMD = −0.64, 95% CI [−1.10, −0.17], *p* = 0.007), indicating a medium effect size. Although the between-study heterogeneity was substantial (I^2^ = 71%), sensitivity analysis revealed that exclusion of any single study did not materially change the pooled estimate, indicating a robust overall effect despite between-study variability.

Ten studies investigated the effects of HIIT on diastolic blood pressure (DBP), including 284 participants. The results showed that HIIT had a significant effect on decreasing DBP in children and adolescents with overweight or obesity (SMD = −0.32, 95% CI [−0.64, −0.01], *p* = 0.045), indicating a small effect size. Between-study heterogeneity was moderate (I^2^ = 43%), and sensitivity analysis revealed that exclusion of any single study did not materially change the pooled estimate, indicating a robust overall effect despite between-study variability.

**Figure 6:**
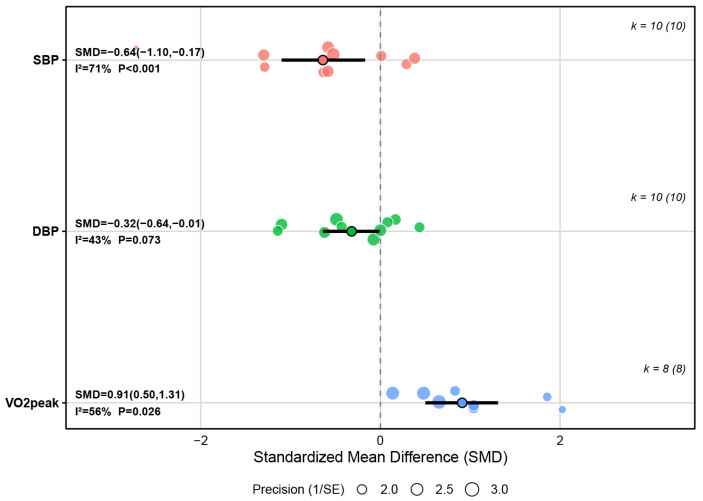
Summary of the impact of HIIT on cardiorespiratory fitness.

Eight studies examined the effects of HIIT on peak oxygen uptake (VO_2_peak) in children and adolescents with overweight or obesity, including 255 participants. The results showed that HIIT had a significant effect on improving VO_2_peak in children and adolescents with overweight or obesity compared with the control conditions (SMD = 0.91, 95% CI [0.50, 1.31], *p* < 0.001), indicating a large effect size. The between-study heterogeneity was moderate (I^2^ = 56%), and sensitivity analysis revealed that exclusion of any single study did not materially change the pooled estimate, indicating a robust overall effect despite between-study variability. More details could be found in Figure 6 and Figure S3 in Supplementary Material S2.

#### 3.4.4. Results of Subgroup Analysis

To examine whether different individual factors and training characteristics may influence intervention outcomes, we selected four representative indicators to perform subgroup analysis: body fat percentage, total cholesterol, HOMA-IR, and peak oxygen uptake. These indicators encompass body composition, lipid metabolism, glucose metabolism, and cardiorespiratory fitness, providing comprehensive coverage and sufficient data for subgroup analyses. The subgroup moderators included sex (female or male), load duration (≤1 min or >1 min), and training mode (running or cycling). The first reflects physiological differences among participants, whereas the latter two capture variations in training intensity and loading characteristics. Based on these indicators and moderators, we conducted subgroup analyses to further investigate whether different training parameters and individual characteristics influence the effectiveness of HIIT interventions in children and adolescents with overweight or obesity. More details of subgroup analyses can be found in Table 1, Table 2, Table 3 and Table 4 and Supplementary Material S3.

#### 3.4.5. Subgroup Analysis for BF%

In the sex subgroup, eight studies comprised samples of female-only participants; the pooled estimate for this subgroup demonstrated a significant reduction in body fat percentage with a medium effect (SMD = −0.67, 95% CI [−1.13,−0.22], *p* < 0.01). Seven studies included only males and revealed a similar outcome (SMD = −0.62, 95% CI [−1.09,−0.15], *p* = 0.01).

In the load duration subgroup analysis, 14 studies with load duration ≤ 1 min (i.e., each high-intensity bout lasted ≤ 1 min) showed a significant reduction in body fat percentage with a medium effect size (SMD = −0.69, 95% CI [−1.03, −0.35], *p* < 0.01). By contrast, four studies with load duration > 1 min did not demonstrate a significant effect (SMD = −0.74, 95% CI [−1.67, 0.20], *p* = 0.12).

In the training mode subgroup, fourteen studies using running as the primary training mode indicated a significant effect on decreasing body fat percentage in children and adolescents with overweight and obesity, with a close-to-large effect size (SMD = −0.76, 95% CI [−1.11, −0.4], *p* < 0.01), whereas three studies employing cycling interventions yielded non-significant results (SMD = −0.67, 95% CI [−1.61, 0.27], *p* > 0.05). More details of the subgroup analyses for BF% are presented in Table 3.

#### 3.4.6. Subgroup Analysis for TC

In the sex subgroup, three studies comprised samples of female-only participants, and the pooled results showed that HIIT had no statistically significant effect on total cholesterol (SMD = −0.28, 95% CI [−0.79, 0.22], *p* > 0.05). Three studies with male-only participants showed a significant reduction in TC with a close-to-large effect (SMD = −0.76, 95% CI [−1.23, −0.28], *p* < 0.01).

In the load duration subgroup, five studies with load duration ≤ 1 min showed a significant reduction in total cholesterol with a medium effect size (SMD = −0.56, 95% CI [−0.90, −0.23], *p* < 0.01). However, four studies with longer load duration (>1 min) showed no significant effect on decreasing TC (SMD = −0.3, 95% CI [−0.73, 0.13], *p* > 0.05).

In the training mode subgroup, five studies using running as the training mode demonstrated a near-significant reduction in total cholesterol (SMD = −0.30, 95% CI [−0.61, 0.02], *p* = 0.06), while two studies employing cycling interventions reported a significant decrease with a large effect size (SMD = −0.99, 95% CI [−1.58, −0.39], *p* < 0.01). More details of the subgroup analyses for TC are presented in Table 4.

#### 3.4.7. Subgroup Analysis for HOMA-IR

In the sex subgroup, five studies comprised female-only samples, and the pooled results showed a significant reduction in HOMA-IR with a large effect size (SMD = −1.08, 95% CI [−1.64, −0.52], *p* < 0.01). Two studies with male-only participants demonstrated a pooled effect that was close to significant (SMD = −1.31, 95% CI [−2.77, 0.14], *p* = 0.07).

In the load duration subgroup, seven studies employing load duration ≤ 1 min showed a significant decrease in HOMA-IR with a large effect size (SMD = −1.26, 95% CI [−1.66, −0.85], *p* < 0.01). In contrast, two studies with load duration > 1 min revealed no statistically significant change (SMD = −0.17, 95% CI [−0.63, 0.30], *p* = 0.48).

In the training mode subgroup, seven studies using running as the training mode indicated a significant reduction in HOMA-IR with a large effect size (SMD = −1.21, 95% CI [−1.68, −0.74], *p* < 0.01). However, among the studies reporting HOMA-IR, only one used cycling as its training mode; therefore, there were insufficient data to determine whether cycling is an effective training mode for improving insulin resistance. More details of the subgroup analyses for HOMA-IR are presented in Table 5.

#### 3.4.8. Subgroup Analysis for VO_2_peak

In the sex subgroup, three studies comprised female-only participants, and the pooled analysis revealed a significant increase in VO_2_peak with a medium effect size (SMD = 0.42, 95% CI [0.03, 0.81], *p* = 0.03). Four studies included male-only samples, and the outcome showed a larger improvement, with a large effect size (SMD = 1.4, 95% CI [0.9, 1.9], *p* < 0.01).

In the load duration subgroup, five studies with load duration ≤ 1 min indicated a significant increase in VO_2_peak with a large effect size (SMD = 0.97, 95% CI [0.55, 1.4], *p* < 0.01). However, three studies with load duration > 1 min reported an outcome that was close to statistically significant (SMD = 0.84, 95% CI [−0.05, 1.73], *p* = 0.06).

In the training mode subgroup, four studies employing running as the primary training mode showed a significant improvement in VO_2_peak, with a medium effect size (SMD = 0.42, 95% CI [0.03, 0.81], *p* = 0.06). Three studies using cycling interventions also demonstrated a significant increase, with a large effect size that was greater than that of running (SMD = 1.27, 95% CI [0.7, 1.85], *p* < 0.01). More details of the subgroup analyses for VO_2_peak are presented in Table 6.

### 3.5. Sensitivity and Publication Bias Analysis

We utilized Egger’s test and funnel plots in Stata 18 to assess publication bias for the 14 outcomes that demonstrated statistically significant pooled effects (*p* < 0.05). The test results indicated potential publication bias for five indicators—BMI, body fat percentage, LDL-C, HOMA-IR, and VO_2_peak (*p* < 0.05)—and the funnel plots of these indicators were also asymmetrical. To address potential publication bias, the trim-and-fill method was applied and the adjusted pooled estimates were recalculated accordingly. The findings showed that the direction and level of statistical significance of these indicators remained unchanged before and after adjustment, suggesting that potential publication bias did not materially affect the overall conclusions.

In addition, sensitivity analyses were conducted for all outcome measures using the leave-one-out method. During this process, each study was sequentially removed, and the pooled effect size was recalculated and compared with the original estimate to assess the influence of individual studies. The results revealed that no single study had a substantial impact on the overall results. This finding showed that all the outcomes were robust, and none of them were altered by any single study, including those with potential risks of bias.

Taken together, these findings indicate that despite the inclusion of studies with relatively small sample sizes or potential sources of bias, the pooled estimates in this meta-analysis were both robust and reliable. For more details of sensitivity and publication bias analysis, please refer to Supplementary Material S4 and S5.

### 3.6. Certainty of Evidence

The GRADE evaluation indicated that the certainty of evidence varied across outcomes. Evidence was rated as high for LBM, TC, DBP, and insulin; moderate for BMI, WC, BF%, FM, TG, HDL-C, LDL-C, glucose, and VO_2_peak; and low for HOMA-IR and SBP. Therefore, the beneficial effects of HIIT on several outcomes are supported by moderate-to-high certainty evidence, whereas findings for HOMA-IR and SBP should be interpreted with greater caution. The details of GRADE evaluation can be found in Table 7.

## 4. Discussion

This meta-analysis, which aimed to evaluate the comprehensive effects of high-intensity interval training (HIIT) on health-related outcomes in children and adolescents with overweight or obesity, synthesized evidence from 21 randomized controlled trials encompassing 652 participants. The main findings demonstrated that HIIT yielded significant improvements across key measures of body composition, metabolic health, and cardiorespiratory fitness in children and adolescents with overweight or obesity. Meanwhile, substantial heterogeneity was observed for several outcomes, indicating meaningful between-study variability. This heterogeneity likely reflects both clinical and methodological differences across trials. With respect to intervention design, HIIT prescriptions were not uniform in terms of training modality, load duration, intensity prescription, work-to-rest structure, or weekly training volume, and these differences may influence energy expenditure, cardiovascular stress, exercise tolerance, and ultimately the magnitude of adaptation. Intervention duration also varied across studies, and shorter programs may be insufficient to induce measurable changes in adiposity or metabolic regulation, whereas longer interventions may allow training adaptations to accumulate more consistently. In addition, participant characteristics were not uniform, with differences in sex composition, age distribution, baseline adiposity, and metabolic status, all of which may affect responsiveness to exercise interventions. Our subgroup analyses partly support this interpretation, as protocols with brief work bouts (≤1 min) tended to yield larger pooled effects for several outcomes, with lower between-study variability in some analyses. Nevertheless, because the number of studies within some subgroups remained limited and reporting of potentially important modifiers was not always sufficiently detailed, these observations should be interpreted cautiously. Notably, our leave-one-out sensitivity analyses did not materially alter the pooled estimates, suggesting that the overall findings were not driven by any single study. Future trials with more standardized intervention reporting and more homogeneous participant profiles are warranted. Concurrently, the GRADE assessment indicated that certainty of evidence differed by outcome, with most outcomes rated moderate to high in certainty, which strengthens the persuasiveness of the main conclusions from a certainty-of-evidence perspective; nevertheless, the lower certainty for some outcomes underscores the need for additional high-quality, methodologically rigorous studies to consolidate and strengthen the evidence base.

### 4.1. Effects of HIIT on Body Composition in Children and Adolescents with Overweight or Obesity

Our meta-analysis found that HIIT produced significant improvements in several main indices of body composition, such as body fat percentage, body mass index, waist circumference, and fat mass. These findings align with those reported by Duncombe, Zhu, and Zheng, further supporting the beneficial role of HIIT on body composition in children and adolescents with overweight or obesity [43,44,45]. While several outcomes improved significantly, HIIT did not yield a significant increase in lean body mass, which may be due to differences in stimulus characteristics: the accrual of skeletal muscle and bone generally requires progressive, weight-bearing resistance loading [46,47], whereas typical running- or cycling-based HIIT provides insufficient mechanical tension. Accordingly, the body composition benefits in this meta-analysis were most evident as reductions in adiposity.

Several mechanisms may account for these effects. Acute exercise imposes substantial demands on glycogen and oxygen, resulting in excess post-exercise oxygen consumption (EPOC) [48]. Meanwhile, exercise stimulus enhances the central neural drive, thereby augmenting sympathetic outflow. This sympathetic activation elevates stress hormones, including catecholamines, which remain above the baseline during early recovery and continue to activate lipolytic signaling via the PKA–HSL/ATGL pathway [49,50,51,52]. The combination of rapid substrate depletion and sustained lipolytic activation shifts post-exercise fuel use from carbohydrate toward fat, thereby increasing fat oxidation [53]. Because the magnitudes of EPOC and recovery fat oxidation are related to exercise intensity, HIIT typically elicits a larger EPOC accompanied by higher post-exercise fat oxidation relative to isoenergetic continuous exercise [54,55,56].

During a long-term intervention period, repeated training stimuli may enhance the body’s capacity for fat oxidation, as reflected by a lower respiratory exchange ratio (RER) and higher fat oxidation at a given exercise intensity, and by increases in maximal fat oxidation (MFO) and its corresponding intensity (Fat_max_) [57,58,59]. This enhancement likely arises because repeated high-intensity training activates pathways such as AMPK/PGC-1α, which promote mitochondrial biogenesis, providing more sites for fat oxidation [60,61]. In parallel, the activities of critical oxidative enzymes involved in lipid metabolism, such as citrate synthase and β-hydroxyacyl-CoA dehydrogenase (β-HAD), are upregulated [62,63], making mitochondrial fatty-acid catabolism more efficient and thereby increasing fat oxidation during exercise.

However, not all previous syntheses have reached the same conclusion. For example, Men et al. reported no significant effect of HIIT on body composition in a meta-analysis of 47 trials [64]. A closer inspection suggests that the inclusion of trials with normal-weight participants may have diluted the effects that are specific to overweight or obese populations. By contrast, our analysis restricted inclusion to trials enrolling children and adolescents with overweight or obesity, and the pooled effects remained robust in sensitivity analyses.

It is worth to notice that body fat assessment is a key predictor of cardiometabolic risk in youth. Numerous studies have shown that in children and adolescents, higher body fat levels are associated with insulin resistance, dyslipidemia, and other components of metabolic syndrome, and that body fat percentage is more effective than BMI for identifying these cardiometabolic risks [65,66,67]. Our subgroup analyses provide further evidence on body fat percentage across different moderators. The results showed that interventions with shorter load duration (≤1 min per high-intensity bout) produced larger reductions, plausibly because shorter bouts allow higher relative intensity, thereby provoking greater catecholamine responses, larger EPOC, and enhanced post-exercise lipid oxidation [54,68]. Despite sex-specific physiological differences during childhood and adolescence, our subgroup analyses showed significant reductions in body fat percentage in both males and females, indicating that HIIT is effective in both genders. When training mode was considered as another moderator, running-based HIIT showed a greater effect on reducing BF% than cycling. This may be because running generally recruits more lower-limb and core musculature, increasing energy expenditure during exercise and thereby producing larger reductions in body fat. However, it is important to acknowledge that empirical evidence on sex- and mode-specific mechanisms is limited, and targeted studies are needed to confirm these hypotheses.

### 4.2. Effects of HIIT on Metabolic Health in Children and Adolescents with Overweight or Obesity

Obesity in children and adolescents can lead to metabolic dysregulation, typically manifested as increased triglyceride (TG) and low-density lipoprotein cholesterol (LDL-C) levels, decreased high-density lipoprotein cholesterol (HDL-C) levels, impaired glucose metabolism, and insulin resistance, all of which are characteristic features of metabolic syndrome [69,70]. These metabolic disturbances are recognized as early risk factors for type 2 diabetes and various cardiovascular diseases (CVDs), such as atherosclerosis [71,72,73].

The findings of this meta-analysis revealed that HIIT effectively improved both lipid metabolism indicators (e.g., triglyceride and cholesterol levels) and glucose metabolism markers (e.g., insulin level and HOMA-IR). These results are consistent with those of Song, Solera-Martínez, and García-Hermoso, all of whom reported the efficacy of HIIT in enhancing metabolic health among children with overweight or obesity [74,75,76].

However, ref. [77] observed no apparent improvement in insulin resistance or related parameters in a two-week small-sample intervention, suggesting that the metabolic benefits of HIIT may depend on a longer intervention duration and greater cumulative training load.

Mechanistically, beyond the energy metabolism and mitochondrial adaptations discussed above, exercise training can enhance the expression of skeletal muscle glucose transporter type 4 (GLUT4), thereby increasing peripheral insulin sensitivity and improving glucose handling [78]. In addition, animal studies have also demonstrated that HIIT can enhance insulin sensitivity in the liver and adipose tissue [79]. Collectively, these findings provide further evidence supporting the role of HIIT in improving metabolic function.

Subgroup analyses indicated that HIIT significantly improved both lipid and glucose metabolism parameters in children and adolescents with overweight or obesity, regardless of sex. In the load duration subgroups, protocols with shorter high-intensity bouts (≤1 min) produced more pronounced improvements and showed lower heterogeneity across studies, suggesting that brief yet intense interval stimuli may be more effective in alleviating metabolic dysregulation among youth with obesity.

Regarding training mode, running-based HIIT elicited greater improvements in total cholesterol and HOMA-IR than cycling-based protocols. However, as the number of cycling studies was limited, further empirical evidence is warranted to confirm these findings.

### 4.3. Effects of HIIT on Cardiorespiratory Fitness in Children and Adolescents with Overweight or Obesity

VO_2_peak serves as a primary indicator of cardiorespiratory fitness (CRF), whereas SBP and DBP reflect arterial pressure and can collectively represent vascular resistance and cardiovascular regulatory capacity. Given that all three parameters are closely associated with circulatory function, this study considered VO_2_peak, SBP, and DBP as cardiorespiratory fitness indices to comprehensively evaluate the influence of HIIT on oxygen transport efficiency and vascular regulation.

The findings revealed that HIIT is an effective intervention for lowering both SBP and DBP in children and adolescents with overweight or obesity, with a more pronounced effect observed for SBP. This aligns with multiple previous studies that have consistently demonstrated HIIT’s efficacy in improving SBP [11,64,80,81]. In contrast, evidence regarding its effect on DBP remains inconsistent. While some studies have reported significant reductions in DBP following HIIT—results consistent with those of the present analysis [64]—others have found no notable changes [11,76,82]. Considering the moderate heterogeneity across studies included in this meta-analysis, the impact of HIIT on DBP requires further confirmation through high-quality randomized trials.

Cardiorespiratory fitness is a key determinant of long-term health in youth, as higher fitness levels during childhood and adolescence are linked to lower risks of disease and all-cause mortality later in life [83,84]. VO_2_peak, as a core measure of CRF, directly reflects the improvement of cardiopulmonary function. Consistent with previous evidence [75,81], our results indicate that HIIT significantly enhances VO_2_peak in children and adolescents with overweight or obesity. Subgroup analyses further revealed that HIIT improved VO_2_peak across both sexes. With respect to load duration, protocols with ≤1 min of high-intensity bouts produced greater improvements, suggesting that exercise intensity is a key factor influencing the effectiveness of HIIT on VO_2_peak. Regarding training mode, both running- and cycling-based HIIT protocols significantly improved VO_2_peak; however, the effect size was greater in cycling interventions, possibly due to their more stable power output and sustained cardiovascular stimulus. These findings suggest that short-duration, high-intensity cycling HIIT may represent a more effective strategy for enhancing cardiorespiratory fitness in children and adolescents with overweight or obesity.

### 4.4. General Remarks and Limitations

Taken together, HIIT simultaneously promotes favorable changes in body composition, metabolic regulation, and cardiorespiratory fitness, suggesting a systemic enhancement in multiple physiological systems in children and adolescents with overweight or obesity. These findings highlight HIIT as an integrated and time-efficient approach capable of reversing key pathophysiological features of pediatric obesity and reinforcing its value as a viable public health strategy for youth populations.

In addition, our subgroup analyses revealed that protocols employing brief work duration (≤1 min) consistently yielded larger pooled effects than those with longer bouts (>1 min) across several outcomes, with a tendency toward lower between-study variability in some cases. This result indicated that HIIT with short bout (≤1 min) might be a more effective way to improve body composition, metabolic health, and cardiorespiratory fitness in children and adolescents with overweight or obesity. Meanwhile, short load duration is able to enhance training adherence by mitigating the physiological burden and exercise monotony of prolonged sessions. Thus, we recommend short load duration as a preferred HIIT protocol.

Despite these strengths, several limitations should be acknowledged. First, most studies did not apply standardized controls for dietary intake, daily physical activity, or sleep patterns, potentially confounding the independent effects of HIIT on body composition and metabolic outcomes.

Second, substantial variability existed across HIIT protocols, including differences in training modality, load duration, intensity prescription, work-to-rest structure, and intervention duration. Such protocol heterogeneity may have contributed to between-study variability and may limit the direct comparability of pooled estimates across trials.

In addition, although the overall number of included studies was acceptable, some individual trials and several subgroup comparisons were based on relatively small sample sizes, which may reduce statistical precision and limit the generalizability of certain findings. Therefore, the present results should be interpreted with appropriate caution, and future studies with larger samples and more standardized HIIT protocols are warranted.

## 5. Conclusions

In conclusion, this meta-analysis provides robust evidence that HIIT yields comprehensive benefits for children and adolescents with overweight or obesity. HIIT effectively improves body composition, metabolic health, and cardiorespiratory fitness, underscoring its strong potential as a practical intervention for this population.

Specifically, HIIT was found to reduce BMI, body fat percentage and waist circumference, indicating a favorable modulation of body composition. It also improved key metabolic parameters—including insulin resistance, total cholesterol, and lipid profiles—and, to some extent, fasting glucose levels. Moreover, HIIT enhanced peak oxygen uptake (VO_2_peak) and reduced systolic blood pressure, suggesting improved cardiorespiratory fitness and vascular regulation.

Moving forward, future studies should further refine HIIT protocols—such as training intensity, work-to-rest ratio and intervention duration—while controlling for confounding factors like diet, habitual activity, and sleep. Incorporating metabolomics and biomarker analyses could deepen understanding of the mechanisms through which HIIT promotes multisystem health. Ultimately, these findings provide a scientific foundation for integrating HIIT into school-based physical education and broader public health initiatives aimed at improving youth fitness and preventing obesity-related diseases. In practical settings, brief and well-supervised HIIT programs may represent a feasible option for obesity management in children and adolescents, particularly when incorporated into school, community, or family-supported exercise environments.

## Figures and Tables

**Table 1:** PICOS framework.

Population	Children and adolescents aged 5–19 years with overweight or obesity, defined according to WHO criteria or corresponding national standards; no restrictions on sex, nationality, or region.
Intervention	High-intensity interval training (HIIT) delivered as a structured exercise intervention, regardless of training modality (e.g., running or cycling), provided that HIIT was the primary intervention component.
Comparison	Control conditions including usual daily activity, no additional exercise intervention, or conventional activity/exercise not meeting the HIIT definition.
Outcome	At least one outcome related to body composition (e.g., BMI, waist circumference, body fat percentage, fat mass, lean body mass), metabolic health (e.g., total cholesterol, triglycerides, HDL-C, LDL-C, glucose, insulin, HOMA-IR), or cardiorespiratory fitness/cardiovascular indicators (e.g., VO_2_peak, systolic blood pressure, diastolic blood pressure).
Study design	Randomized controlled trials published in English or Chinese with extractable quantitative data.

**Table 2:** Basic characteristics of the included studies.

Included Studies	Sample Ratio (T/C)	Sex	Age	Duration (Weeks)	Frequency (Days/Week)	Protocol	Outcome Measures
Abassi et al., 2023 [22]	13/12	Female	15–18	12	3	2 sets of 6 to 8 repetitions of 30 s 100–110% MAS running + 30 s recovery at 50% MAS running; rest between sets: 4 min	BMI, WC, BF%
Abassi et al., 2020 [23]	8/8	Female	16.5 ± 1.36	12	3	2 sets of 6 repetitions of 30 s 100–110% MAS running + 30 s recovery at 50% MAS running; rest between sets: 4 min	BMI; WC; BF%; INS; GLU
Abassi et al., 2025 [24]	17/16	Female	17 ± 1.15	9	3	2 sets of 6 to 8 repetitions of 30 s 100–105% MAS running + 30 s recovery at 50% MAS running; rest between sets: 4 min	BMI, WC, BF%, TC, HDL-C, LDL-C, TG, INS, GLU, HOMA-IR, SBP, DBP
Ahmadi et al., 2020 [25]	28/27	Mixed	10–16	8	3	9 sets of 3 min aerobic training + 20 s recovery	BMI, WC, TC, HDL-C, LDL-C, TG
Alizadeh and Safarzade, 2019 [26]	10/10	Male	18 ± 1.5	6	3	4–6 sets of 30 s maximum shuttle run + 30 s recovery	BMI, BF%
Cao et al., 2022 [27]	17/18	Mixed	11 ± 0.8	12	3	3 bouts of 8 repetitions of 15 s 100–120% MAS running + 15 s recovery at 50% MAS; rest between sets: 3 min	BMI, WC, BF%, FM, LBM, TC, HDL-C, LDL-C, TG, INS, GLU, HOMA-IR, SBP, DBP
Chuensiri et al., 2018 [28]	11/11	Male	8–12	12	3	8 sets of 2 min 90% peak power output cycling + 1 min recovery	BMI, WC, BF%, TC, HDL-C, LDL-C, TG, SBP, DBP, VO_2_peak
Cvetković et al., 2018 [29]	11/14	Male	11–13	12	3	3 sets of 5 repetitions of 10–20 s 100% MAS running with 1:1 passive recovery; rest between sets: 3 min	BMI, BF%, FM, LBM, SBP, DBP
Dias et al., 2018 [30]	17/21	Mixed	7–16	12	3	4 sets of 4 min 85–95% HRmax training + 3 min recovery of 50–70% HRmax training	BMI, BF%, FM, LBM, TC, HDL-C, LDL-C, TG, GLU, HOMA-IR, VO_2_peak
González-Gálvez et al., 2024 [31]	11/12	Mixed	12.51 ± 0.75	8	2	3 sets of 120 s 80–85% RHR running with 120 s active recovery at 50–55% RHR running	BMI, BF%, LBM, SBP, DBP
Kou et al., 2025 [32]	19/20	Male	HIIT: 14.7 ± 1.3 Control: 14.2 ± 1.2	16	3	15 sets of 30 s 90% VO_2max_ cycling + 30 s 40% VO_2max_ cycling	BMI, BF%, FM, LBM
Meng et al., 2022 [33]	12/13	Male	11–13	12	3	2 times of 8 repetitions of 15 s 90–100% MAS running + 15 s recovery at 50% MAS; rest between sets: 5 mins	BMI, WC, BF%, FM, LBM, TC, HDL-C, LDL-C, TG, INS, GLU, HOMA-IR, SBP, DBP, VO_2_peak
Meng et al., 2022 [34]	20/20	Male	11 ± 0.6	12	3	3 sets of 8 repetitions of 15 s 100% MAS running + 15 s recovery at 50% MAS; rest between sets: 3 mins	BMI, BF%, FM, LBM
Plavsic et al., 2020 [35]	22/22	Female	15.8 ± 1.6	12	2	4 sets of 4 min 85–90% HRmax running with 3 min recovery at 70% HRmax	BMI, WC, BF%, LBM, HDL-C, LDL-C, TG, TC, HOMA-IR, SBP, DBP, VO_2_peak
Racil et al., 2013 [36]	11/12	Female	15.9 ± 0.3	12	3	2 sets of 6 repetitions of 30 s 100–110%MAS running + 30 s recovery of 50% MAS; rest between sets: 4 min	WC, BF%, TC, HDL-C, LDL-C, TG, INS, GLU, HOMA-IR, VO_2_peak
Racil et al., 2016 [37]	17/14	Female	14.2 ± 1.2	12	3	3 sets of 8–16 repetitions of 15 s 100% MAS running + 15 s 50% MAS recovery; rest between sets: 3 min	WC, BF%, INS, GLU, HOMA-IR, SBP, DBP
Racil et al., 2016 [39]	23/19	Female	16.6 ± 1.3	12	3	2 sets of 6–8 repetitions of 30 s running at 100% velocity at VO_2_peak + 30 s recovery at 50% velocity at VO_2_peak; rest between sets: 4 min	WC, BF%, LBM, INS, GLU, HOMA-IR, VO_2_peak
Racil et al., 2024 [38]	12/11	Female	14.4 ± 1.4	8	3	4 sets of 6 repetitions of 15 s running at (90–105)% MAS + 15 s recovery at 50% MAS; rest between sets: 3 min	WC, BF%, GLU, SBP, DBP
Salus et al., 2022 [40]	14/14	Male	12–16	12	3	4–6 sets of 30 s all-out cycling (Wingate tests) + 4 min active recovery (cycling at 30 W) between each set	WC, BF%, FM, LBM, SBP, DBP, VO_2_peak
Salus et al., 2022 [41]	14/14	Mixed	12–16	12	3	4–6 repetitions of 30 s all-out cycling bouts interspersed with 4 min active rest after each about	TC, HDL-C, LDL-C, TG, INS, GLU, HOMA-IR, VO_2_peak
Salus et al., 2023 [42]	18/19	Male	13.4 ± 0.3	12	3	4–6 sets of 30 s cycling with 4 mins recovery between each set	BMI

T: Experimental Group; C: Control Group; MAS: Maximum Aerobic Speed; HRmax: Maximum Heart Rate; VO_2max_: Maximal Oxygen Uptake; BMI: Body Mass Index; WC: Waist Circumference; BF%: Body Fat Percentage; FM: Fat Mass; LBM: Lean Body Mass; TC: Total Cholesterol; HDL-C: High-Density Lipoprotein Cholesterol; LDL-C: Low-Density Lipoprotein Cholesterol; TG: Triglycerides; INS: Insulin; GLU: Blood Glucose; HOMA-IR: Homeostatic Model Assessment of Insulin Resistance; SBP: Systolic Blood Pressure; DBP: Diastolic Blood Pressure; VO_2_peak: Peak Oxygen Uptake.

**Table 3:** Subgroup analysis results for BF%.

Outcomes	Subgroups	No. of Studies	Heterogeneity Test Results		Effects Models	Meta-Analysis Results	
			*p*	I^2^		95% CI	*p*
BF%	**Sex**						
	Female	8	<0.01	63%	Random	−0.67 [−1.13, −0.22]	<0.01
	Male	7	0.02	61%	Random	−0.62 [−1.09, −0.15]	0.01
	**Load duration**						
	≤1 min	14	<0.01	62%	Random	−0.69 [−1.03, −0.35]	<0.01
	>1 min	4	<0.01	82%	Random	−0.74 [−1.67, 0.2]	0.12
	**Training mode**						
	Running	14	<0.01	65%	Random	−0.76 [−1.11, −0.4]	<0.01
	Cycling	3	0.01	78%	Random	−0.67 [−1.61, 0.27]	0.16

**Table 4:** Subgroup analysis results for TC.

Outcomes	Subgroup	No. of Studies	Heterogeneity Test Results		Effects Models	Meta-Analysis Results	
			*p*	I^2^		95% CI	*p*
TC	**Sex**						
	Female	3	0.22	34%	Random	−0.28 [−0.79, 0.22]	>0.05
	Male	3	0.43	0%	Random	−0.76 [−1.23, −0.28]	<0.01
	**Load duration**						
	≤1 min	5	0.7	0%	Random	−0.56 [−0.9, −0.23]	<0.01
	>1 min	4	0.16	41%	Random	−0.3 [−0.73, 0.13]	0.17
	**Training mode**						
	Running	5	0.77	0%	Random	−0.3 [−0.61, 0.02]	0.06
	Cycling	2	0.3	17%	Random	−0.99 [−1.58, −0.39]	<0.01

**Table 5:** Subgroup analysis results for HOMA-IR.

Outcomes	Subgroup	No. of Studies	Heterogeneity Test Results		Effects Models	Meta-Analysis Results	
			*p*	I^2^		95% CI	*p*
HOMA-IR	**Sex**						
	Female	5	0.02	65%	Random	−1.08 [−1.64, −0.52]	< 0.01
	Male	2	0.2	81%	Random	−1.31 [−2.77, 0.14]	0.07
	**Load duration**						
	≤1 min	7	0.09	45%	Random	−1.26 [−1.66, −0.85]	<0.01
	>1 min	2	0.39	0	Random	−0.17 [−0.63, 0.30]	0.48
	**Training mode**						
	Running	7	0.01	61%	Random	−1.21 [−1.68, −0.74]	<0.01
	Cycling	1	N/A	N/A	Random	−0.61 [−1.37, 0.15]	0.1

**Table 6:** Subgroup analysis results for VO2peak.

Outcomes	Subgroup	No. of Studies	Heterogeneity Test Results		Effects Models	Meta-Analysis Results	
			*p*	I^2^		95% CI	*p*
VO_2_peak	**Sex**						
	Female	3	0.01	62%	Random	0.42 [0.03, 0.81]	0.03
	Male	4	0.29	20%	Random	1.4 [0.9, 1.9]	<0.01
	**Load duration**						
	≤1 min	5	0.21	31%	Random	0.97 [0.55, 1.40]	<0.01
	>1 min	3	0.01	78%	Random	0.84 [−0.05, 1.73]	0.06
	**Training mode**						
	Running	4	0.03	67%	Random	0.75 [0.1, 1.4]	0.02
	Cycling	3	0.28	23%	Random	1.27 [0.7, 1.85]	<0.01

**Table 7:** GRADE criteria for certainty of evidence on body composition, metabolic health, and cardiorespiratory fitness.

Outcomes	Certainty Assessment							No. of Patients		Effect Size (Hedges’ g, 95% CI)	Certainty
	No. of Studies	Study Design	Risk of Bias	Inconsistency	Indirectness	Imprecision	Other Considerations	HIIT	Control		
**Body Composition**											
**BMI**	15	Randomized trial	Not serious	Not serious	Not serious	Not serious	Publication bias strongly suspected	217	241	−1.05 (−1.56 to −0.55)	⨁ ⨁ ⨁ ◯ Moderate
**WC**	13	Randomized trial	Not serious	Serious	Not serious	Not serious	None	202	195	−0.38 (−0.61 to −0.16)	⨁ ⨁ ⨁ ◯ Moderate
**BF%**	18	Randomized trial	Not serious	Not serious	Not serious	Not serious	Publication bias strongly suspected	263	260	−0.69 (−1.01 to −0.37)	⨁ ⨁ ⨁ ◯ Moderate
**FM**	7	Randomized trial	Not serious	Serious	Not serious	Serious	Strong association	111	114	−0.89 (−1.59 to −0.19)	⨁ ⨁ ⨁ ◯ Moderate
**LBM**	10	Randomized trial	Not serious	Not serious	Not serious	Not serious	None	164	166	−0.03 (−0.24 to 0.19)	⨁ ⨁ ⨁ ⨁ High
**Metabolic health**											
**TC**	9	Randomized trial	Not serious	Not serious	Not serious	Not serious	None	148	150	−0.42 (−0.66 to −0.17)	⨁ ⨁ ⨁ ⨁ High
**TG**	9	Randomized trial	Not serious	Not serious	Not serious	Serious	None	148	150	−0.32 (−0.59 to −0.05)	⨁ ⨁ ⨁ ◯ Moderate
**HDL-C**	9	Randomized trial	Not serious	Not serious	Not serious	Serious	None	148	150	0.45 (0.08 to 0.81)	⨁ ⨁ ⨁ ◯ Moderate
**LDL-C**	9	Randomized trial	Not serious	Not serious	Not serious	Not serious	Publication bias strongly suspected	148	150	−0.58 (−0.94 to −0.21)	⨁ ⨁ ⨁ ◯ Moderate
**Insulin**	8	Randomized trial	Not serious	Not serious	Not serious	Serious	Strong association	119	114	−1.58 (−2.09 to −1.08)	⨁ ⨁ ⨁ ⨁ High
**Glucose**	10	Randomized trial	Not serious	Not serious	Not serious	Serious	None	156	147	−0.53 (−0.96 to −0.09)	⨁ ⨁ ⨁ ◯ Moderate
**HOMA-IR**	10	Randomized trial	Not serious	Serious	Not serious	Serious	Publication bias strongly suspected; strong association	149	141	−1 (−1.46 to −0.54)	⨁ ⨁ ◯◯ Low
**Cardiorespiratory fitness**											
**SBP**	10	Randomized trial	Not serious	Serious	Not serious	Serious	None	119	117	−0.64 (−1.1 to −0.17)	⨁ ⨁ ◯◯ Low
**DBP**	10	Randomized trial	Not serious	Not serious	Not serious	Not serious	None	119	117	−0.32 (−0.64 to −0.01)	⨁ ⨁ ⨁ ⨁ High
**VO2peak**	8	Randomized trial	Not serious	Not serious	Not serious	Serious	Publication bias strongly suspected; strong association	143	147	0.91 (0.5 to 1.31)	⨁ ⨁ ⨁ ◯ Moderate

Notes: CI: confidence interval; SMD: standardized mean difference; ⨁: a filled level of certainty; ◯: an unfilled level of certainty.

## Data Availability

As this study is a systematic review, all data analyzed are available in the main manuscript and the Supplementary Materials. No new datasets were generated. Further information is available from the corresponding author upon reasonable request.

## Supplementary Materials

The following supporting information can be downloaded at https://www.mdpi.com/article/10.3390/metabo16040232/s1. Supplementary Material S1: Searching strategy; Supplementary Material S2: Forest plots of all outcomes; Supplementary Material S3: Forest plots of subgroup analyses; Supplementary Material S4: Funnel plots of all outcomes; Supplementary Material S5: Sensitivity analysis plot of all outcomes; Supplementary Material S6: PRISMA checklist.

## Abbreviations

HIIT, High-Intensity Interval Training; BMI, Body Mass Index; WC, Waist Circumference; BF%, Body Fat Percentage; FM, Fat Mass; LBM, Lean Body Mass; TC, Total Cholesterol; HDL-C, High-Density Lipoprotein Cholesterol; LDL-C, Low-Density Lipoprotein Cholesterol; TG, Triglycerides; INS, Insulin; GLU, Blood Glucose; HOMA-IR, Homeostasis Model Assessment of Insulin Resistance; SBP, Systolic Blood Pressure; DBP, Diastolic Blood Pressure; VO_2_peak, Peak Oxygen Uptake.
