# Supplementary material for: Effects of High-Intensity Interval Training on Body Composition, Metabolic Health, and Cardiorespiratory Fitness in Overweight or Obese Children and Adolescents: A Systematic Review and Meta-Analysis
Source: Metabolites. 2026 Mar 31;16(4):232. doi: 10.3390/metabo16040232 (PMC13117705; doi:10.3390/metabo16040232)
Supplement: Supplementary file 1 [file metabolites-16-00232-s001.zip › S1 Searching strategy.pdf]

Support information S1 Search strategy for **Web of Science, EBSCO One-step, PubMed, CNKI , Wanfang Data and VIP** databases.

Search strategy for **Web of Science** database (inception-September 09, 2025).

**Category**	**Search terms**
Population	#**1** ALL=(children OR childhood OR child* OR adolescent OR youth OR pediatrics)<br>#**2** ALL=(overweight OR obese OR obesity OR obesity OR adiposity OR "excess weight" OR "excess body weight" OR "excess adiposity")<br>#**3** #1 AND #2
AND<br>Intervention	#**4** ALL=("high intensity interval training" OR "high-intensity interval training" OR "high intensity interval exercise" OR "high-intensity interval exercise" OR "high-intensity intermittent training" OR "high intensity intermittent training" OR "high intensity intermittent exercise" OR "high-intensity intermittent exercise" OR "aerobic interval training" OR "aerobic-interval training" OR "aerobic interval exercise" OR "aerobic-interval exercise" OR "interval training" OR "interval exercise" OR "sprint interval training" OR "sprint interval exercise" OR "sprint training")
Result	#**5** #**3 AND** #**4** 243

Search strategy for **EBSCO One-step** database (inception-September 09, 2025).

Category	Search terms
Population	#**1** ALL=(children OR childhood OR child* OR adolescent OR youth OR pediatrics)<br>#**2** ALL=(overweight OR obese OR obesity OR obesity OR adiposity OR "excess weight" OR "excess body weight" OR "excess adiposity")<br>#**3** #1 AND #2
AND<br>Intervention	#**4** ALL=("high intensity interval training" OR "high-intensity interval training" OR "high intensity interval exercise" OR "high-intensity interval exercise" OR "high-intensity intermittent training" OR "high intensity intermittent training" OR "high intensity intermittent exercise" OR "high-intensity intermittent exercise" OR "aerobic interval training" OR "aerobic-interval training" OR "aerobic interval exercise" OR "aerobic-interval exercise" OR "interval training" OR "interval exercise" OR "sprint interval training" OR "sprint interval exercise" OR "sprint training")
Result	#**5** #**3 AND** #**4** 307

Search strategy for **PubMed** database (inception-September 09, 2025).

Category	Search terms
Population	#**1** (((((((("child"[MeSH Terms])) OR ("child"[All Fields])) OR ("children"[All Fields])) OR ("child s"[All Fields])) OR ("childhood"[All Fields] OR "childhoods"[All Fields])) OR ("child*"[All Fields])) OR (((((("adolescent"[MeSH Terms])) OR ("adole*"[All Fields])) OR ("adolescent"[All Fields])) OR ("adolescence"[All Fields])) OR ("adolescents"[All Fields])) OR ((("youth"[All Fields])) OR ("youths"[All Fields])) OR ((((("paediatrics"[All Fields])) OR ("pediatrics"[MeSH Terms])) OR ("pediatrics"[All Fields])) OR ("pediatric"[All Fields])) #**2** (((((((((("Obesity"[MeSH Terms])) OR ("Overweight"[MeSH Terms])) OR ("Pediatric Obesity"[MeSH Terms])) OR ("obes*"[All Fields])) OR ("obese"[All Fields])) OR ("overweigh*"[All Fields])) OR ("excess weight"[All Fields])) OR ("excess body weight"[All Fields])) OR ("adiposity"[All Fields])) OR ("excess adiposity"[All Fields]) #**3** #**1 AND** #**2**
AND Intervention	#**4** ((((((((("high-intensity interval training"[All Fields])) OR ("high-intensity interval training"[All Fields])) OR ("high-intensity interval exercise"[All Fields])) OR ("high-intensity interval exercise"[All Fields])) OR ("high-intensity intermittent training"[All Fields])) OR ("high-intensity intermittent training"[All Fields])) OR ("high-intensity intermittent exercise"[All Fields])) OR ("high-intensity intermittent exercise"[All Fields])) OR (HIIT[MeSH Terms])
Result	#**5** #**3 AND** #**4** 209

Search strategy for **CNKI** database (inception-September 09, 2025).

Category	Search terms
Population	**#1** SU=(adolescent + youth + children + childhood + pediatrics)<br>**#2** SU=(overweight + obese + overweight + overweight + excess adiposity + **central obesity** + **abdominal obesity** + High body fat + excess adiposity)<br>**#3 #1 AND #2**
AND Intervention	**#4** SU=(high-intensity interval exercise + high-intensity interval exercise + high-intensity intermittent training + high-intensity intermittent training + high-intensity intermittent exercise + high-intensity intermittent exercise + Interval sprint training + Sprint interval training)
Result	**#5 #3 AND #4** 40

Search strategy for **Wanfang Data and VIP** databases (inception-September 09, 2025).

Category	Search terms
Population	**#1** ALL=("adolescent" OR "youth" OR "children" OR "childhood" OR "pediatrics")<br>**#2** ALL=("overweight " OR "obesity" OR "overweight " OR "excess weight" OR "obesity" OR "**central obesity** " OR "**abdominal obesity** " OR "High body fat " OR "excess adiposity")<br>**#3 #1 AND #2**
AND Intervention	**#4** ALL=(high-intensity interval exercise + high-intensity interval exercise + high-intensity intermittent training + high-intensity intermittent training + high-intensity intermittent exercise + high-intensity intermittent exercise + Interval sprint training + Sprint interval training)
Result	**#5 #3 AND #4** Wanfang Data (n=47) VIP (n=50)
