# Supplementary material for: Effects of High-Intensity Interval Training on Body Composition, Metabolic Health, and Cardiorespiratory Fitness in Overweight or Obese Children and Adolescents: A Systematic Review and Meta-Analysis
Source: Metabolites. 2026 Mar 31;16(4):232. doi: 10.3390/metabo16040232 (PMC13117705; doi:10.3390/metabo16040232)
Supplement: Supplementary file 1 [file metabolites-16-00232-s001.zip › S2 Forest plots of all outcomes.pdf]

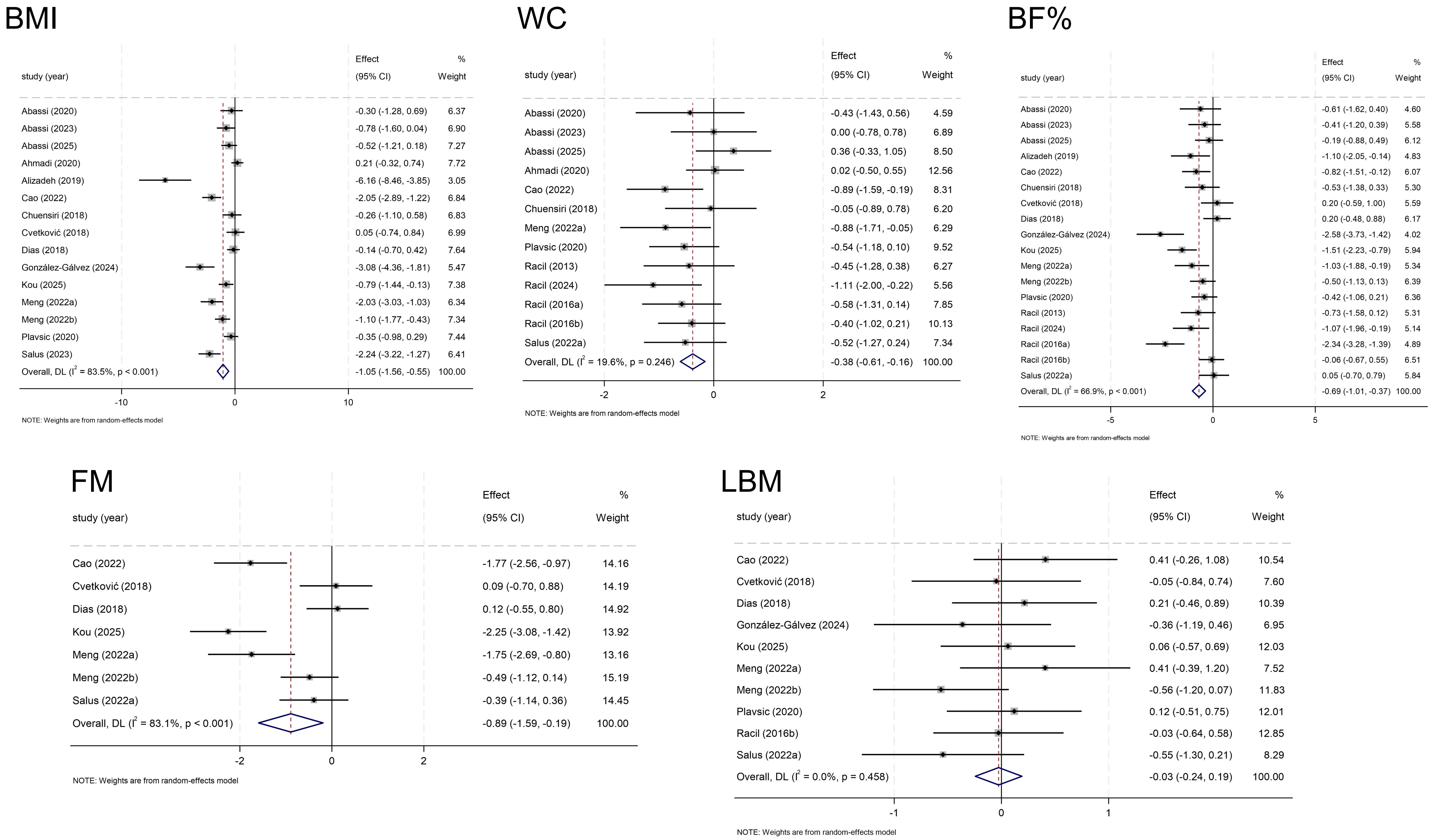

Fig S1.Forest plots of body composition

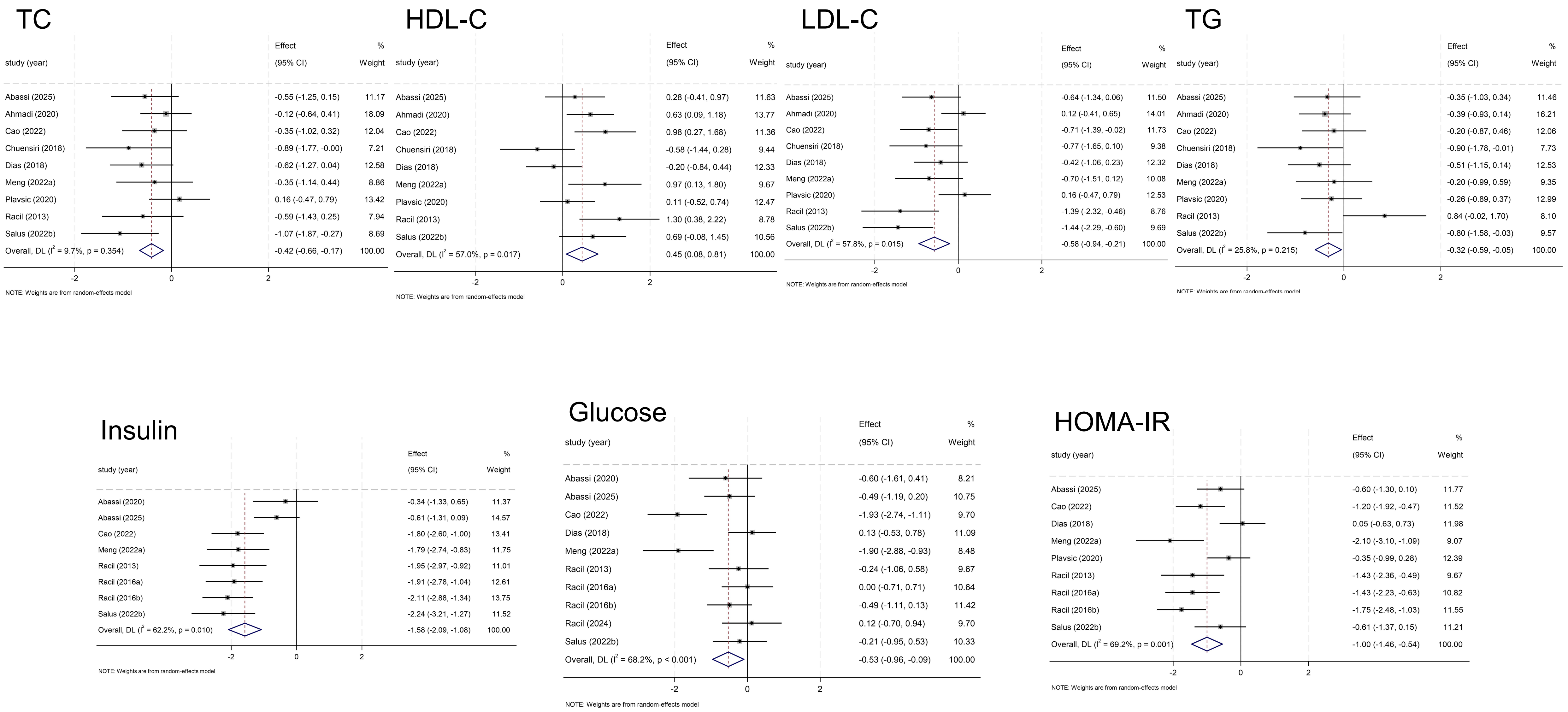

Fig S2.Forest plots of metabolic health

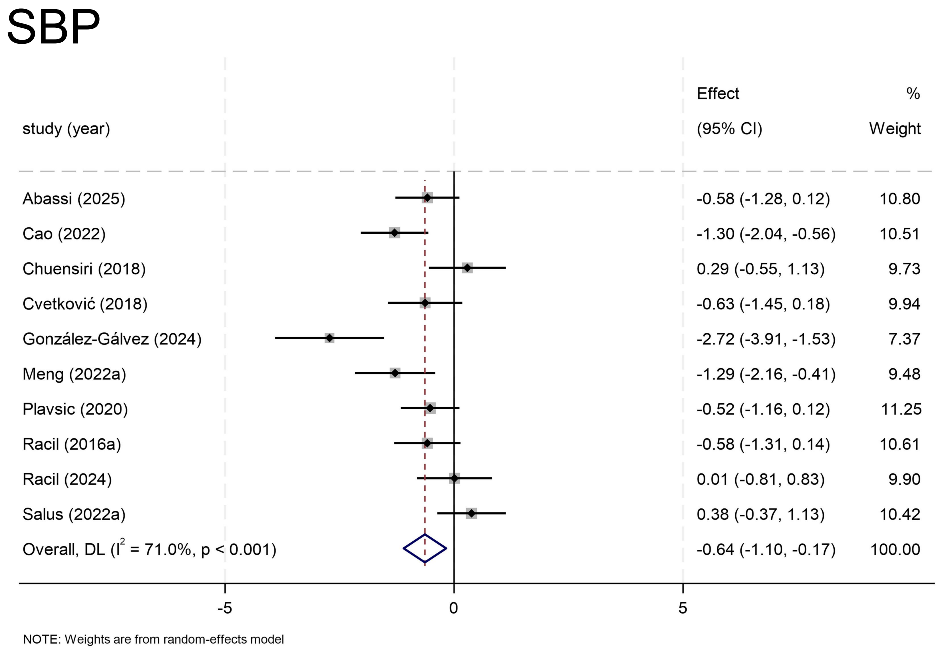

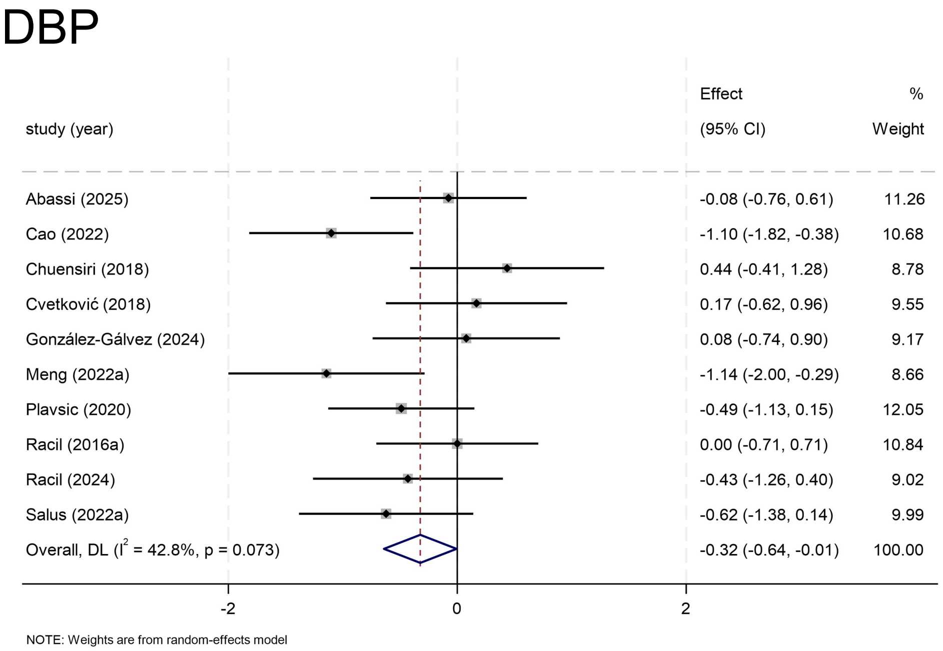

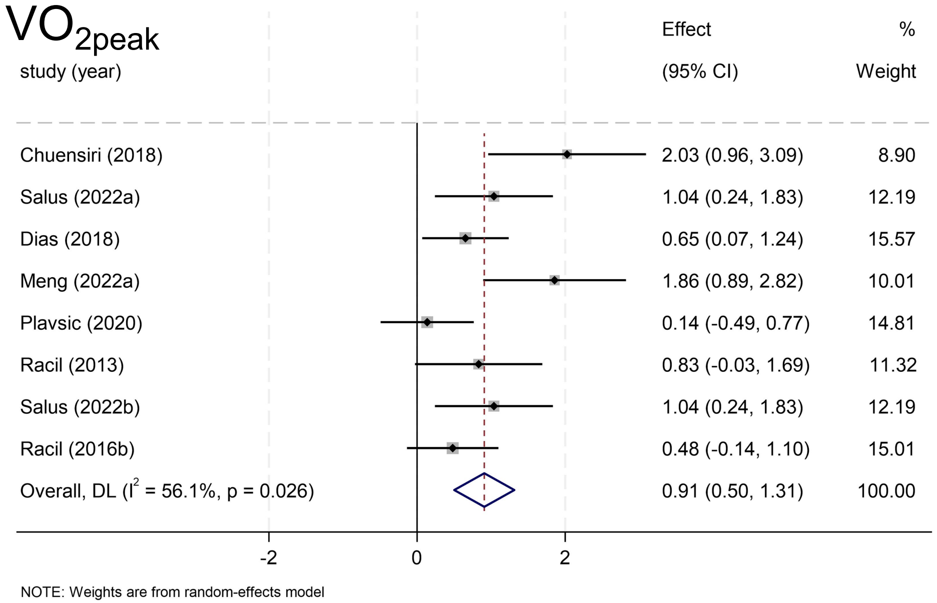

Fig S3.Forest plots of cardiorespiratory fitness
