# Supplementary material for: Effects of High-Intensity Interval Training on Body Composition, Metabolic Health, and Cardiorespiratory Fitness in Overweight or Obese Children and Adolescents: A Systematic Review and Meta-Analysis
Source: Metabolites. 2026 Mar 31;16(4):232. doi: 10.3390/metabo16040232 (PMC13117705; doi:10.3390/metabo16040232)

Subgroup analyses of BF%

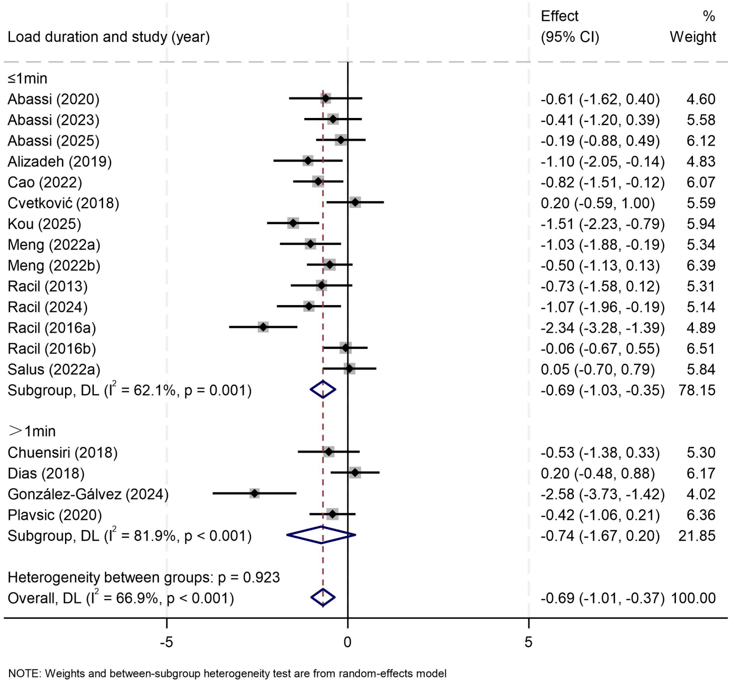

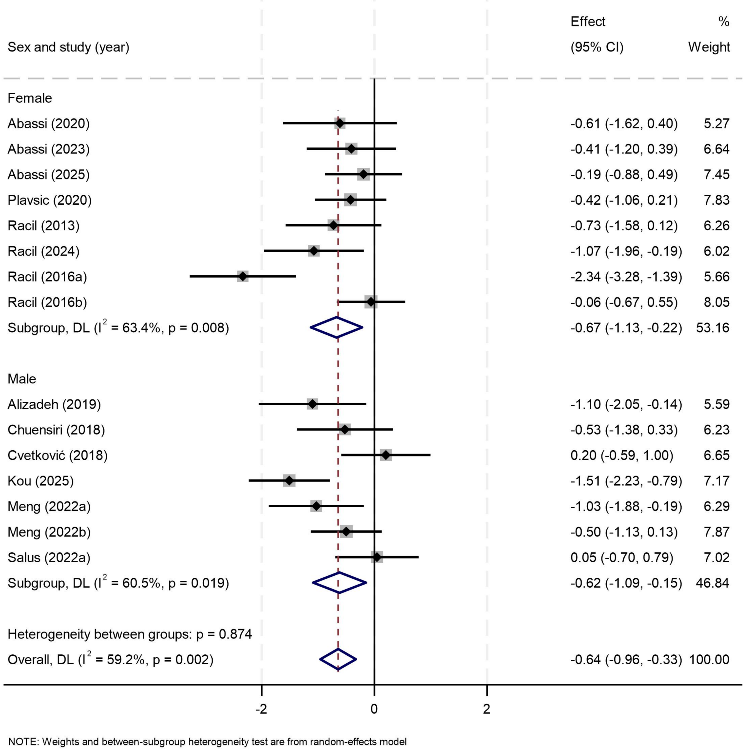

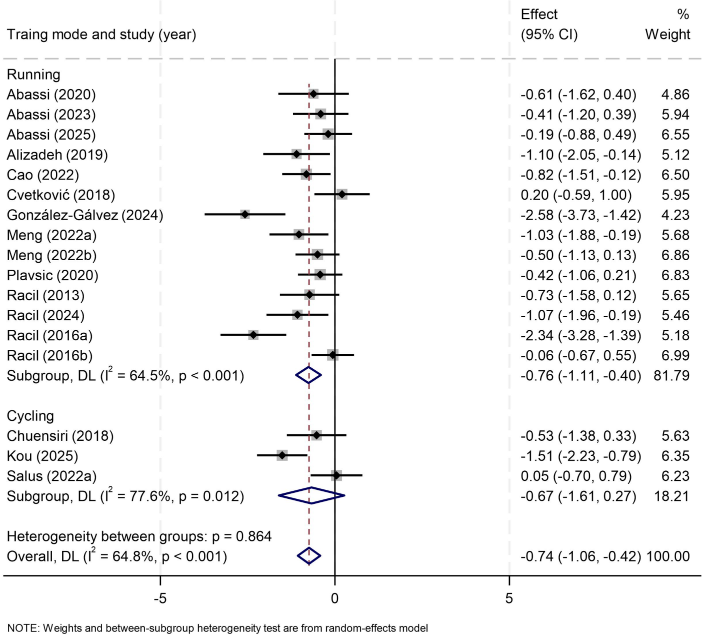

Subgroup analyses of TC

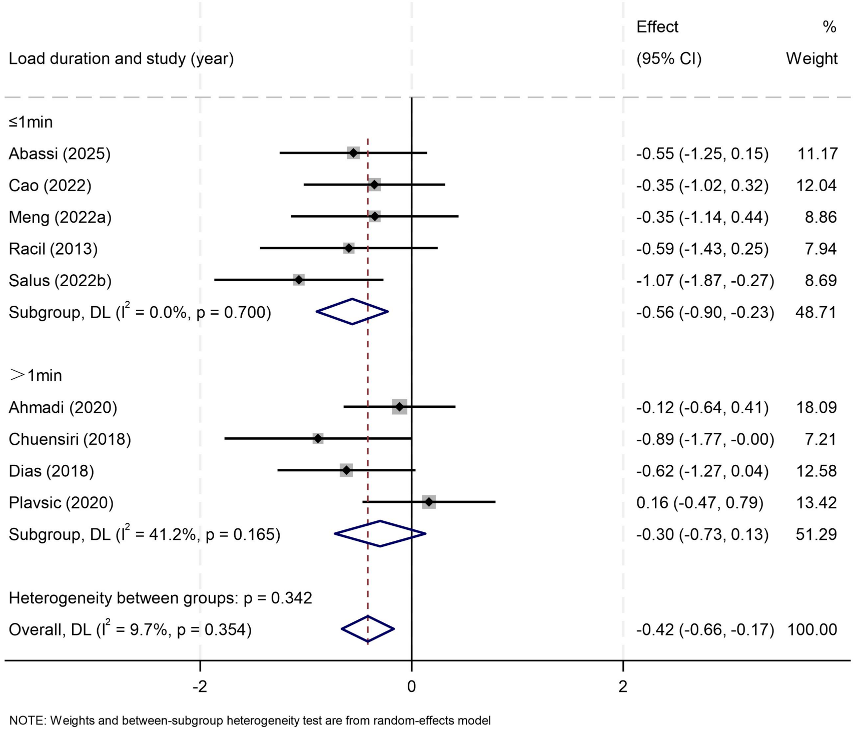

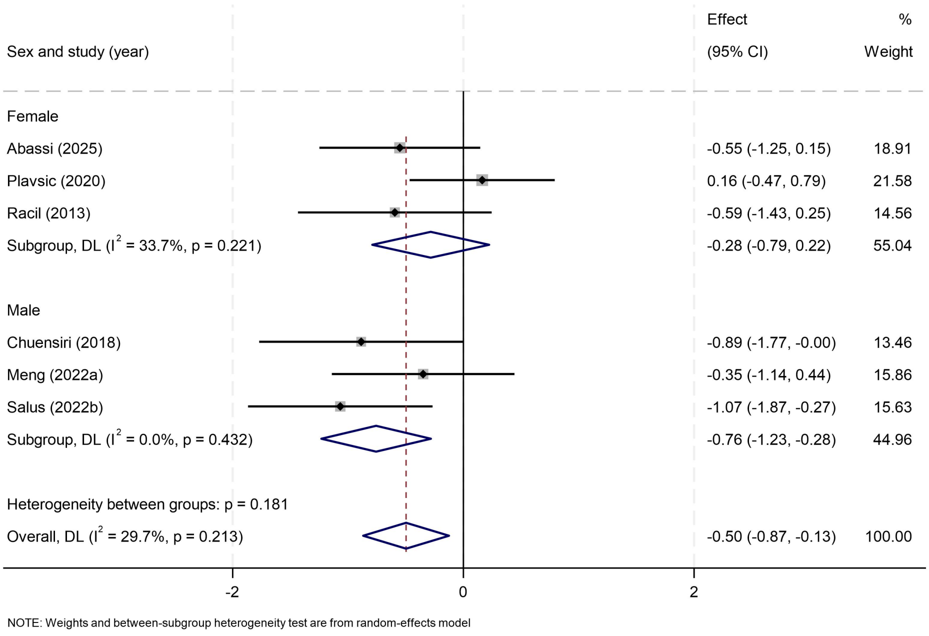

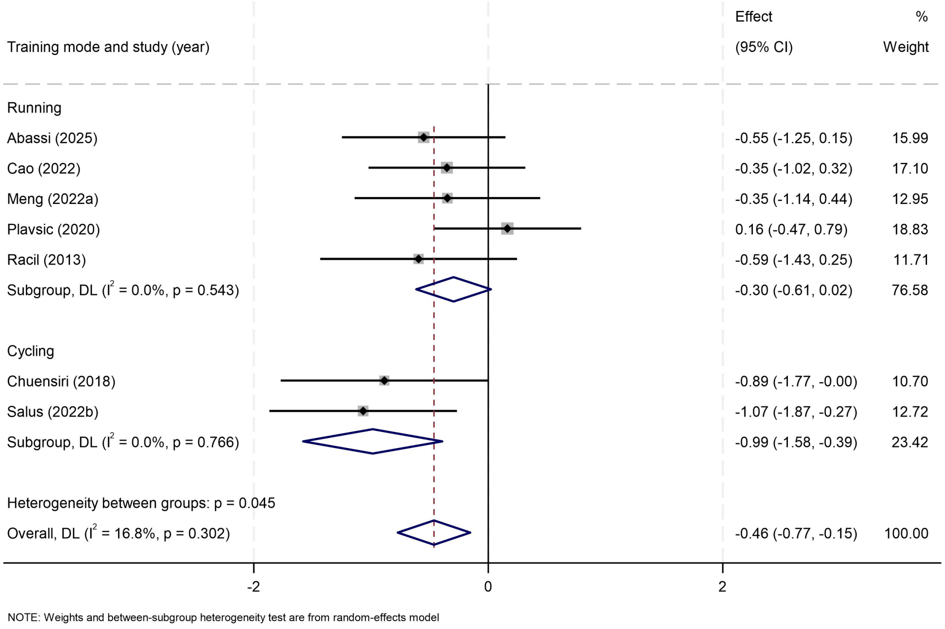

Subgroup analyses of HOMA-IR

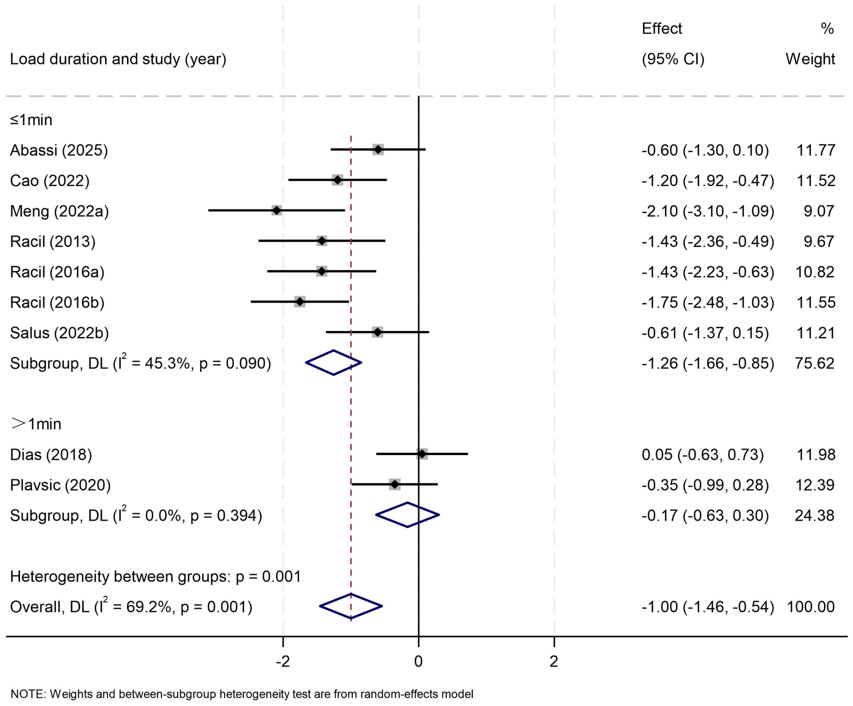

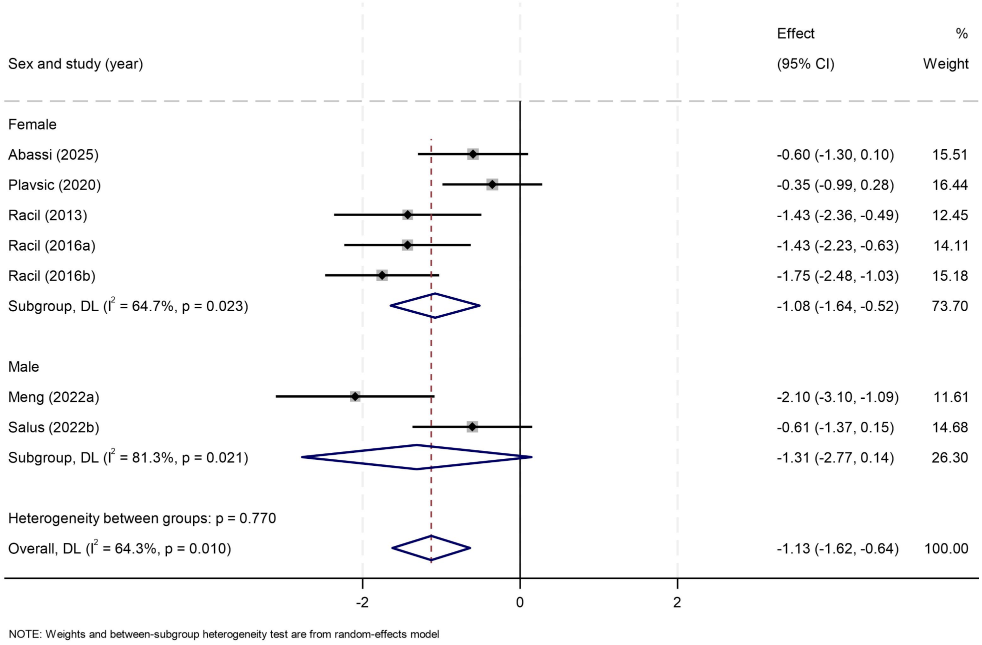

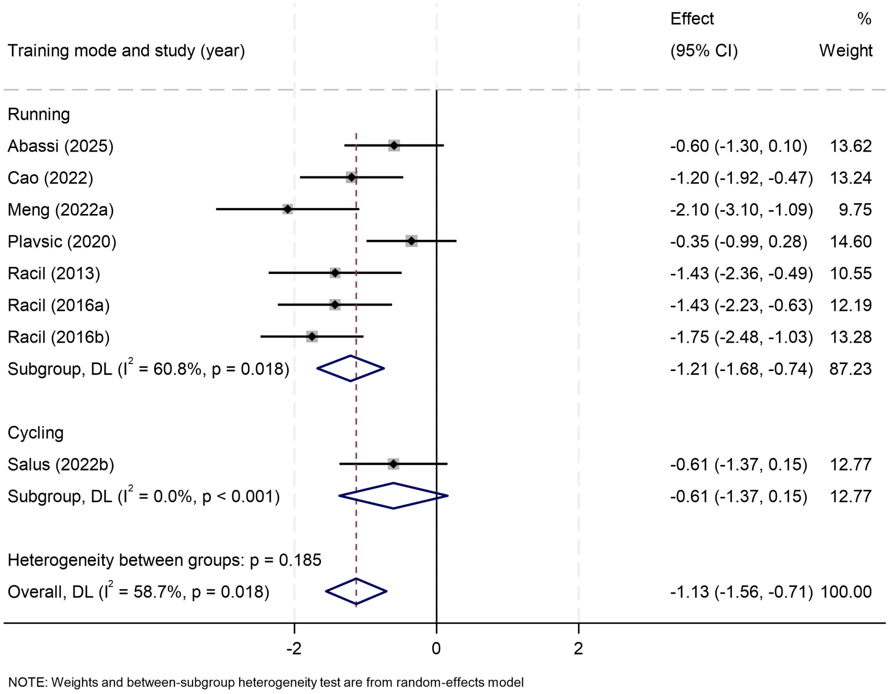

Subgroup analyses of VO_{2peak}

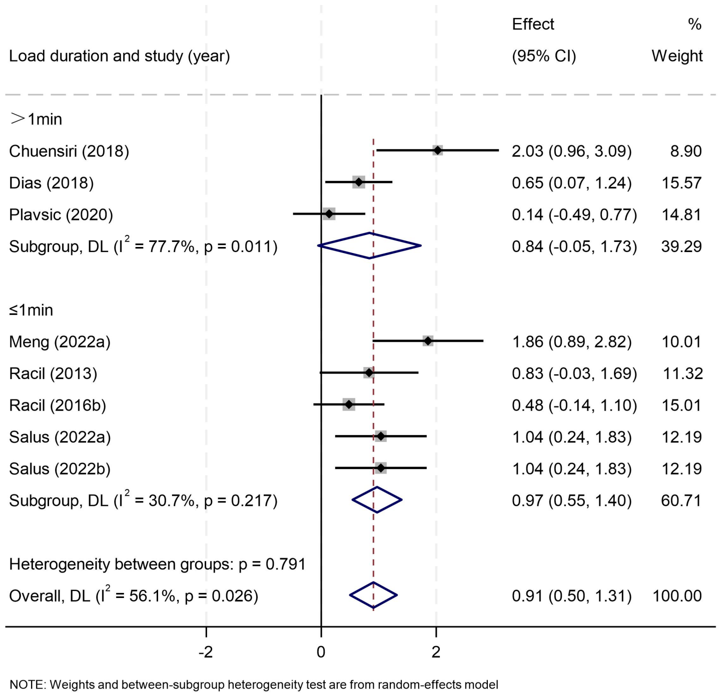

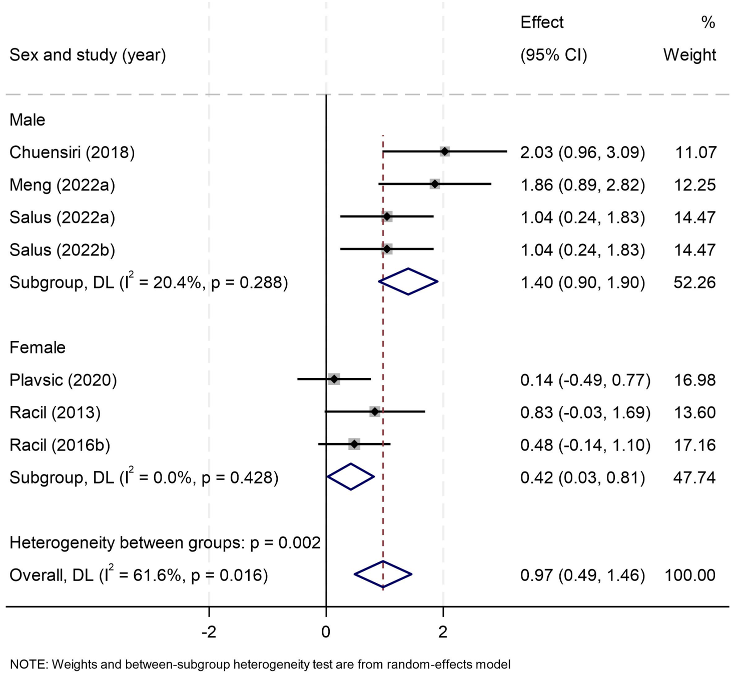

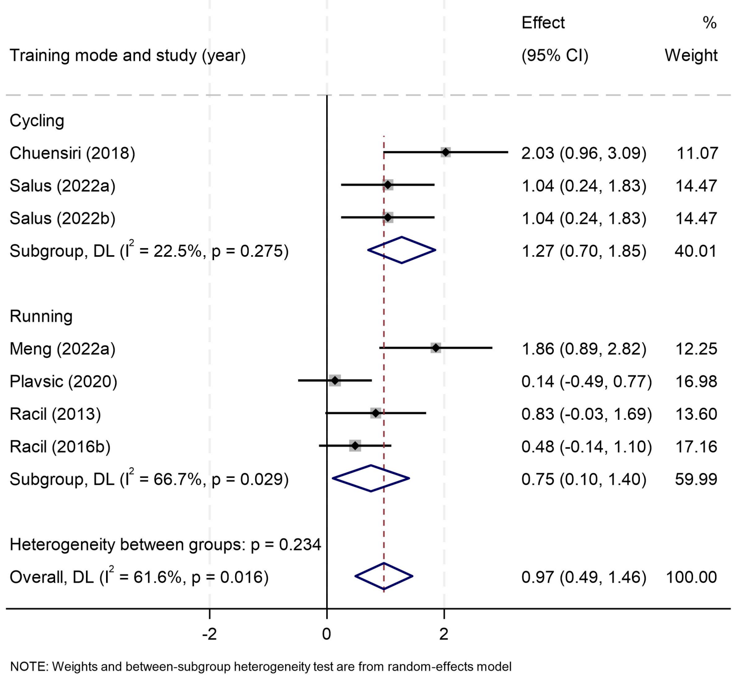

Supplement: Supplementary file 1 [file metabolites-16-00232-s001.zip › S3 Forest plots of subgroup analyses.pdf]
