# Supplementary figures and images for: Effects of High-Intensity Interval Training on Body Composition, Metabolic Health, and Cardiorespiratory Fitness in Overweight or Obese Children and Adolescents: A Systematic Review and Meta-Analysis
Source: Metabolites. 2026 Mar 31;16(4):232. doi: 10.3390/metabo16040232 (PMC13117705; doi:10.3390/metabo16040232)

BMI

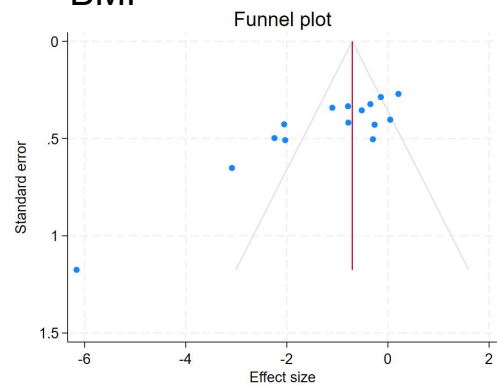

WC

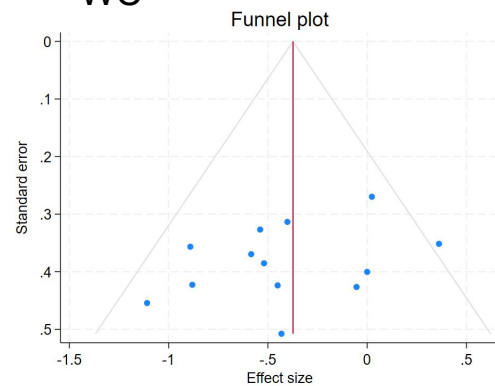

BF%

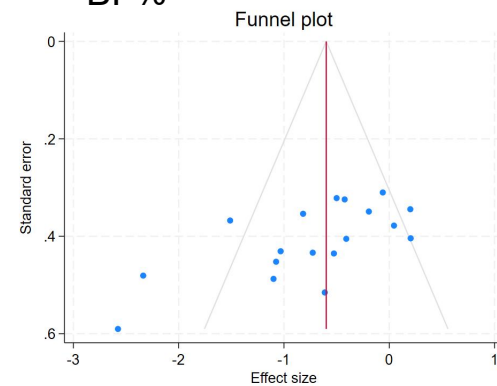

FM

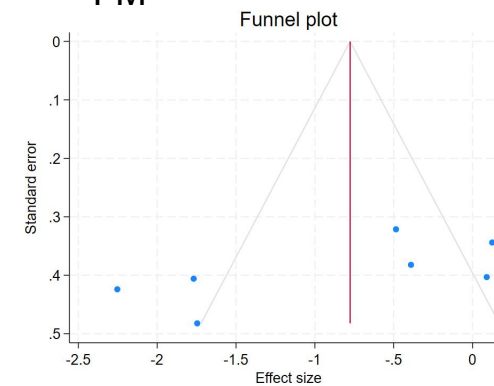

LBM

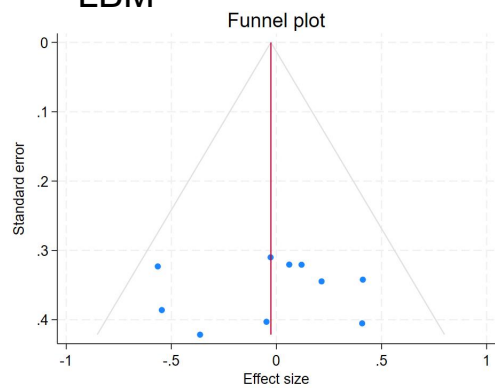

TC

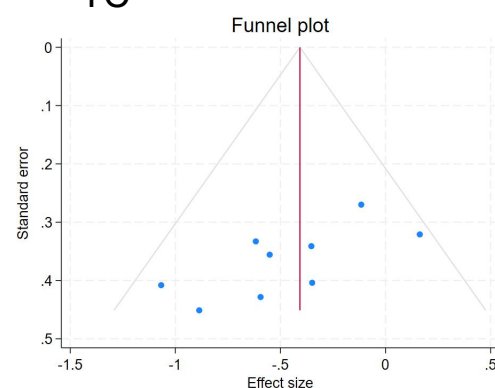

HDL-C

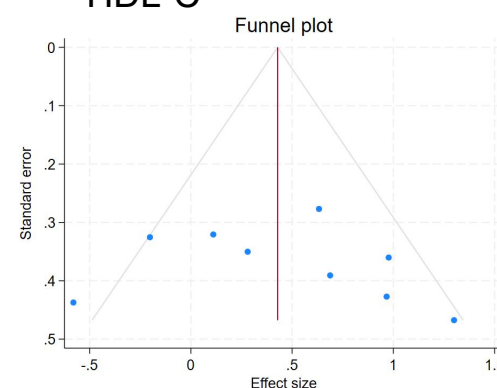

LDL-C

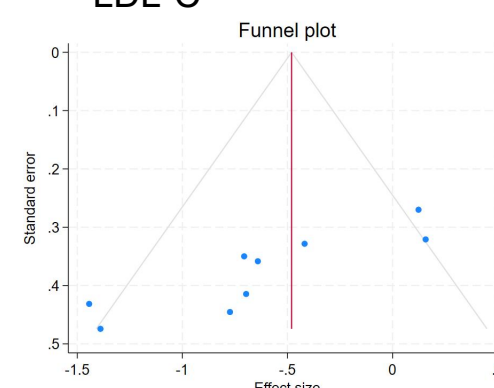

TG

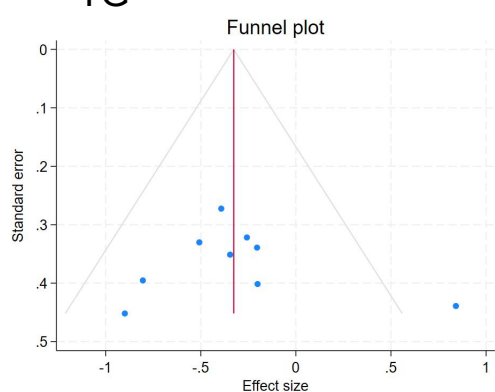

Insulin

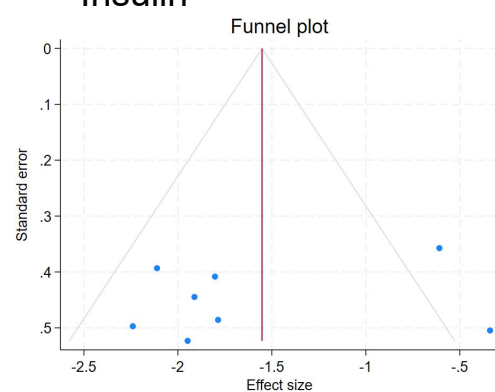

Glucose

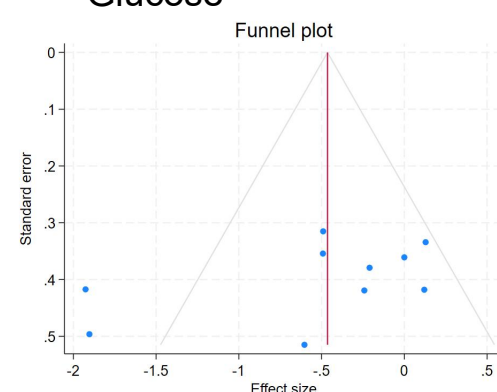

HOMA-IR

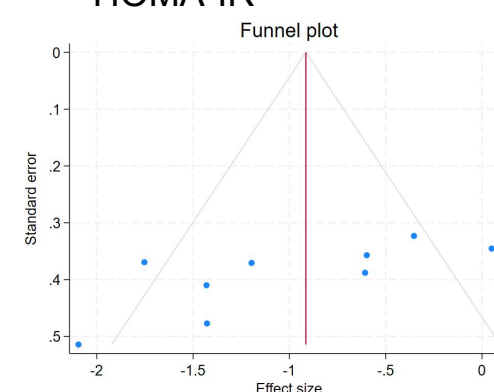

SBP

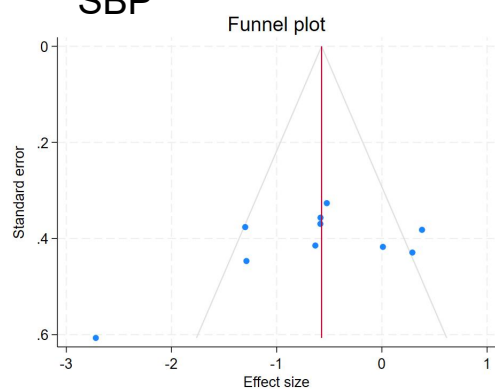

DBP

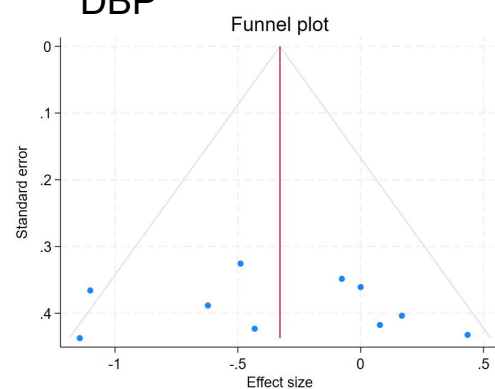VO<sub>2</sub>PEAK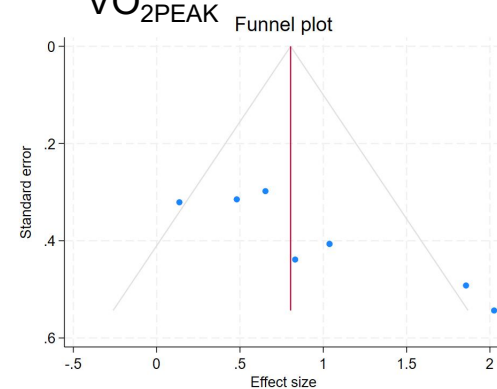

Supplement: Supplementary file 1 [file metabolites-16-00232-s001.zip › S4 Funnel plots of all outcomes.pdf]

BMI

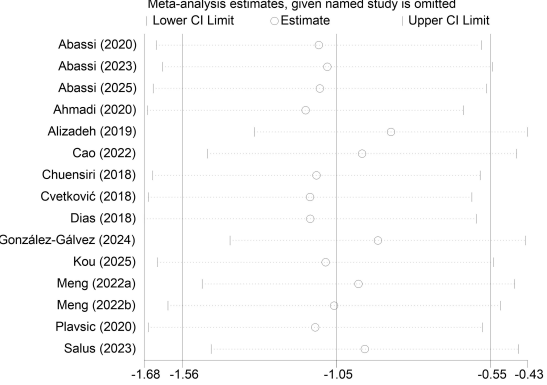

WC

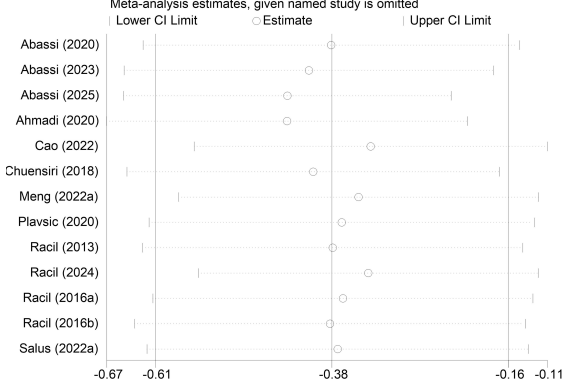

BF%

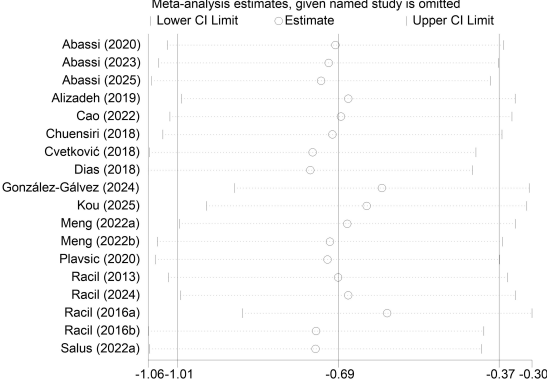

FM

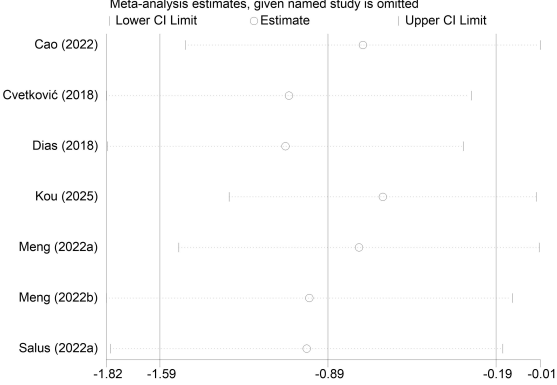

TC

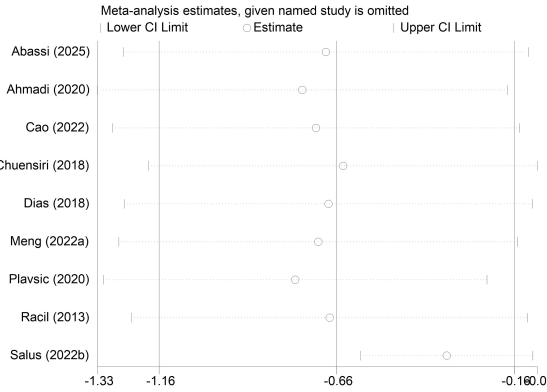

HDL-C

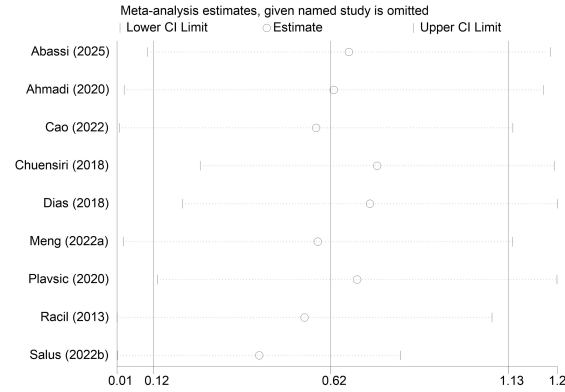

LDL-C

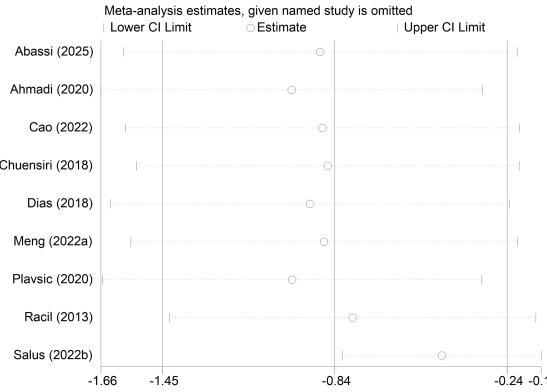

TG

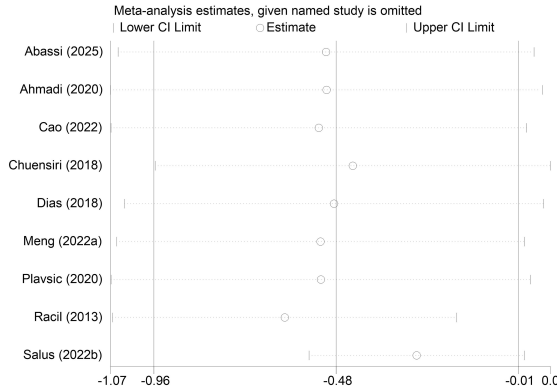

Insulin

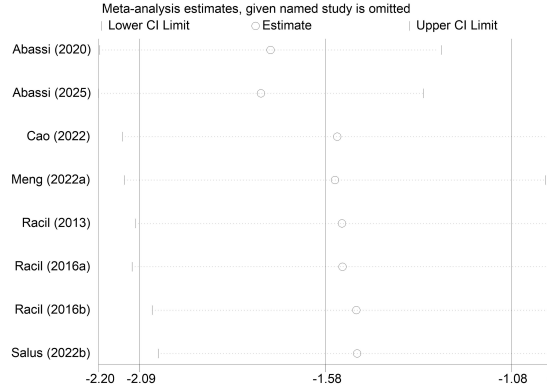

Glucose

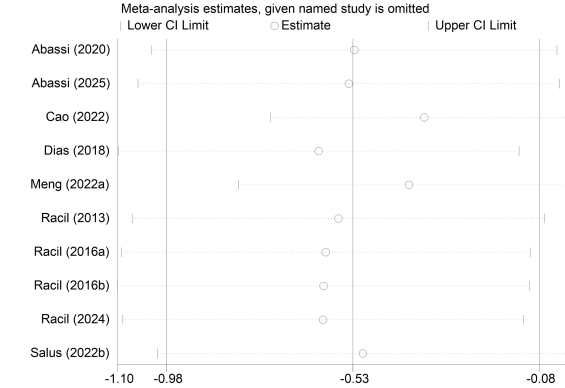

HOMA-IR

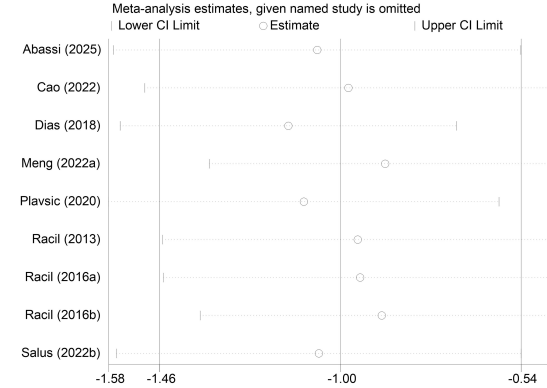

SBP

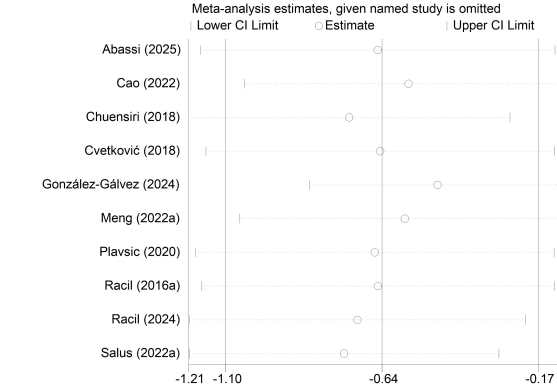

DBP

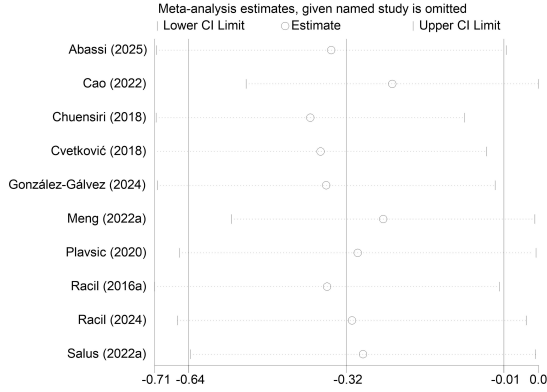

VO<sub>2</sub>peak

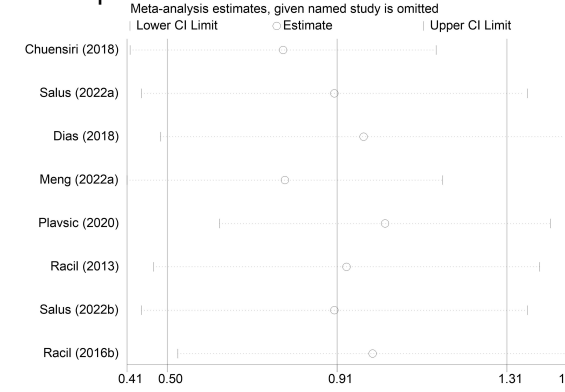

Supplement: Supplementary file 1 [file metabolites-16-00232-s001.zip › S5 Sensitivity plots.pdf]
